# Detection and validation of single feature polymorphisms using RNA expression data from a rice genome array

**DOI:** 10.1186/1471-2229-9-65

**Published:** 2009-05-29

**Authors:** Sung-Hyun Kim, Prasanna R Bhat, Xinping Cui, Harkamal Walia, Jin Xu, Steve Wanamaker, Abdelbagi M Ismail, Clyde Wilson, Timothy J Close

**Affiliations:** 1 Department of Botany and Plant Sciences, University of California, Riverside, CA 92521 USA; 2 Department of Statistics, University of California, Riverside, CA 92521 USA; 3 International Rice Research Institute, Manila, Philippines; 4 United States Department of Agriculture Agricultural Research Service, George E Brown Jr, Salinity Laboratory, Riverside, CA 92507 USA

## Abstract

**Background:**

A large number of genetic variations have been identified in rice. Such variations must in many cases control phenotypic differences in abiotic stress tolerance and other traits. A single feature polymorphism (SFP) is an oligonucleotide array-based polymorphism which can be used for identification of SNPs or insertion/deletions (INDELs) for high throughput genotyping and high density mapping. Here we applied SFP markers to a lingering question about the source of salt tolerance in a particular rice recombinant inbred line (RIL) derived from a salt tolerant and salt sensitive parent.

**Results:**

Expression data obtained by hybridizing RNA to an oligonucleotide array were analyzed using a statistical method called robustified projection pursuit (RPP). By applying the RPP method, a total of 1208 SFP probes were detected between two presumed parental genotypes (Pokkali and IR29) of a RIL population segregating for salt tolerance. We focused on the *Saltol *region, a major salt tolerance QTL. Analysis of FL478, a salt tolerant RIL, revealed a small (< 1 Mb) region carrying alleles from the presumed salt tolerant parent, flanked by alleles matching the salt sensitive parent IR29. Sequencing of putative SFP-containing amplicons from this region and other positions in the genome yielded a validation rate more than 95%.

**Conclusion:**

Recombinant inbred line FL478 contains a small (< 1 Mb) segment from the salt tolerant parent in the *Saltol *region. The Affymetrix rice genome array provides a satisfactory platform for high resolution mapping in rice using RNA hybridization and the RPP method of SFP analysis.

## Background

A SFP is a polymorphism detected by a single probe in an oligonucleotide array [1]. SFPs represent SNPs, INDELs or both. A polymorphism within a transcribed sequence might reflect a biologically pertinent variation within the encoded protein or a regulatory element located in an untranslated region. Therefore, SFPs detected using oligonucleotide microarrays designed for expression analysis can provide function-associated genetic markers.

We initially developed the RPP method of SFP discovery using the Affymetrix barley genome array [2] and then applied this method to rice [3]. A distinguishing component of our method is the use of complex RNA as a surrogate for rice genomic DNA, eliminating genome size and interference from highly repetitive DNA as technical impediments to SFP detection. Another distinguishing element of our method is that RPP first utilizes a probe set level analysis to identify SFP-containing probe sets and then chooses only the one or two most discriminatory probes from within each SFP-containing probe set.

SFPs have been identified using oligonucleotide microarrays in several species. In yeast [4] and Arabidopsis [1], SFPs were detected by hybridization of genomic DNA to oligonucleotide microarrays. SFP genotyping was accomplished also by hybridization of mRNA to an oligonucleotide-expression array in yeast [5]. More recently, SFPs were identified in rice using hybridization of genomic DNA to an oligonucleotide microarray [6,7].

Here we analyzed RNA expression data using the RPP method to detect SFPs among a salt-tolerant rice recombinant inbred line (RIL), FL478, and its presumed parental rice genotypes, Pokkali and IR29, as described previously [2,3]. FL478 was developed from an *indica *cross between salt-tolerant Pokkali and salt-susceptible IR29 [8-10]. Gregorio et al. (1997) identified salt-tolerant and salt-sensitive RILs [9]. One of the RILs, FL478 (F2-derived F8) was among the most salt tolerant.

Our purpose in the present study was to apply higher density SFP analysis to a lingering question about the nature of salt tolerance in RIL FL478, following our previous report that the only SFP markers that we were aware of in the vicinity of the *Saltol *locus in FL478 originated from the salt sensitive parent.

## Results and discussion

### SFP detection and validation

**Figure 1:**
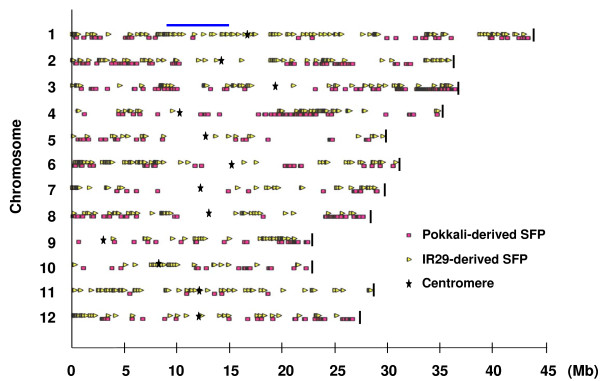
**Rice pseudomolecule map showing positions of SFPs detected in this study**. SFPs in FL478 detected as Pokkali or IR29 haplotype by RPP method are shown in squares (pink) and triangles (yellow), respectively. Stars and vertical bars indicate the positions of the centromeres and the ends of chromosomes, respectively. Horizontal bar (blue) means the *Saltol *region.

**Figure 2:**
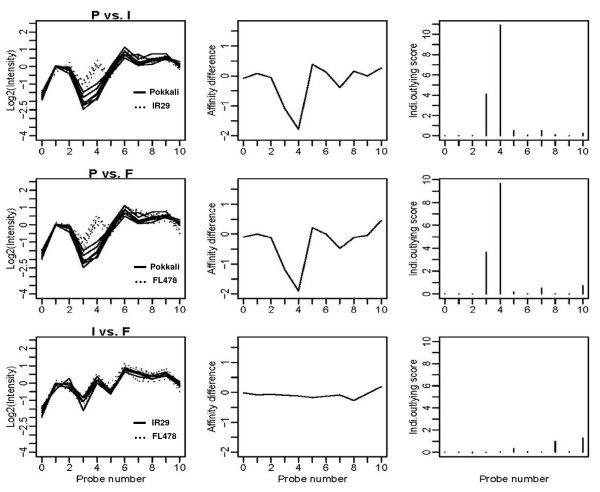
**SFP detection in a probe set by RPP method**. (Left panel) Plots of the log intensities (PM, perfect match) for the representative probe set (Os.33510.1.S2_at) from three genotypes. (Middle panel) Plots of the differentiations of average log intensities among three genotypes. (Right panel) Plots of individual outlying scores. P, Pokkali; I, IR29; F, FL478. After Cui et al. (2005) [2].

**Figure 3:**
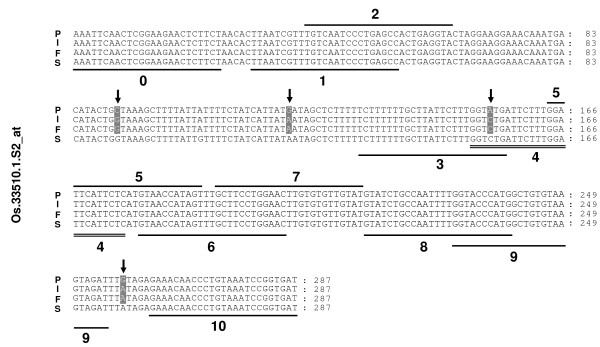
**Nucleotide sequence alignment of amplicon sequences of a probe set**. Polymorphic residues are highlighted in gray. Bars 0–10 indicate the positions of eleven probes in the probe set (Os.33510.1.S2_at). The position of SFP probe number 4 detected by the RPP method is double-underlined. Arrows indicate SNPs. P, Pokkali; I, IR29; F, FL478; S, target sequence from SIF.

By applying higher density SFP analysis than previously, a total of 1208 SFP probes were detected in the present analysis (Figure 1, Additional file 1). Plots of the log intensities, affinity differences and individual outlying scores for a representative probe set (Os.33510.1.S2_at) are shown in Figure 2. The intensity differentiation between Pokkali and FL478 is highest at probes 4 and 3, indicating polymorphism at these probe positions. A representative alignment of the amplicon sequences with the target sequence of Os.33510.1.S2_at probe set is shown in Figure 3. Several SNPs were detected, but only probe positions 3 and 4 span a SNP. Probe 4 was selected as a SFP by the RPP method based on a higher outlying score than that of probe 3 (Figure 2).

### SFPs detected in *Saltol *region by RPP method

**Figure 4:**
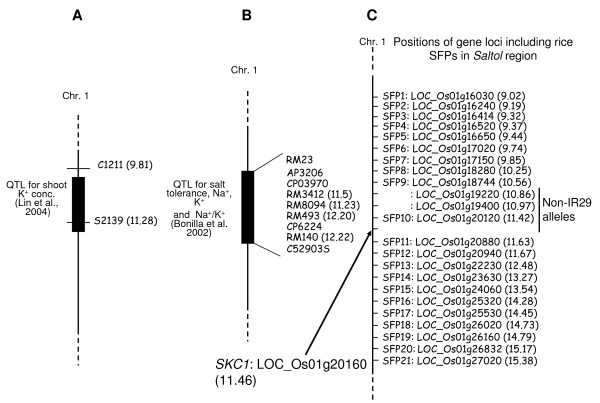
**Chromosome 1 segment associated with a major QTL for salt tolerance**. Genetic linkage maps showing the location of *Saltol *described by Lin et al. (2004) [11] and Bonilla et al. (2002) [8] are shown in (A) and (B), respectively. (C) The segment of pseudomolecule map showing the physical positions of the *SKC1 *gene [12] and loci with SFPs in the *Saltol *region. Numbers in parentheses indicate physical positions (Mb) on chromosome 1.

We explored the source of the *Saltol *region in FL478 because several reports demonstrated the importance of this region for salt tolerance, and because our prior report [3] suggested that the *Saltol *region of FL478 may have originated from the salt sensitive parent. Bonilla et al. (2002) [8] initially delimited *Saltol *as a QTL controlling three traits (low Na^+ ^absorption, high K^+ ^absorption and low Na^+^/K^+ ^ratio) within a 15 cM segment of the rice genetic map with peak LOD score > 6.7 (Figure 4). A major QTL for high shoot K^+ ^concentration under salt stress also was identified in the same region [11]. More recently, Ren et al. (2005) identified the *SKC1 *gene encoding a sodium transporter and demonstrated that it is a determinant of salt tolerance in the *Saltol *region [12].

In prior work we reported that all of the SFPs detected in the *Saltol *region of FL478 were consistent with an IR29 origination (salt sensitive parent) [3], indicating either that FL478 received its salt tolerance from other QTL or that we did not have sufficient SFP marker density in this region to detect a small region of the genome from the salt tolerant parent. Subsequent to the Walia et al. (2005) work [3], we extended the list of SFPs to examine the *Saltol *region in more detail. This was accomplished by: 1) considering all probe sets including those with "_s", "_x" or "_a" in the probe set name in order to give higher SFP density and 2) updating the gene model annotations available from . An explanation of these suffixes is in the Affymetrix GeneChip design manual, which is available from the Affymetrix website. The suffix "_at" at the end of every probe set means antisense transcript. A lack of another suffix means that all probes in the probe set are unique to the particular sequence used for the array design. The "x" indicates that at least one probe is a perfect match to another sequence. The "a" indicates that all probes are a perfect match to another sequence in the same gene family and the "s" indicates that all probes are a perfect match to a sequence in another gene family.

These actions revealed additional SFPs in the *Saltol *region, increasing the total to 21 SFPs among which one corresponding to gene model LOC_Os01g20120 was identical to the Pokkali allele (Table 1, Figure 4), not IR29. This gene model is adjacent to the *SKC1 *gene (LOC_Os01g20160) which as stated above is known to be a salt tolerance gene [12].

**Table 1:** Rice SFP probe sets in the *Saltol *region

Probe set name	Gene model ^a^	Position of 5' end	Annotation ^b^	E-value	IR29 ^c^	Sequenced
Os.35495.1.S1_s_at	LOC_Os01g16030	9020854	Putative ADP-ribosylation factor protein	1.00E-101	+	NO
Os.455.1.S1_at	LOC_Os01g16240	9192919	Putative calmodulin protein	7.00E-81	+	YES
Os.37639.1.S1_at	LOC_Os01g16414	9320117	Actin family protein	0	+	YES
Os.37842.1.S1_at	LOC_Os01g16520	9374978	Glutamyl-tRNA synthetase family protein	0	+	YES
Os.247.1.S1_at	LOC_Os01g16650	9442463	Putative ubiquitin-conjugating enzyme X protein	1.00E-109	+	YES
Os.14702.1.S1_a_at	LOC_Os01g17020	9746901	Expressed protein	1.00E-136	+	YES
Os.7948.1.S1_a_at	LOC_Os01g17150	9856128	Expressed protein	2.00E-74	+	YES
Os.29809.2.S1_x_at	LOC_Os01g18280	10259724	SNF7 family protein	0	+	NO
Os.3655.1.S1_at	LOC_Os01g18744	10562090	Transferase family protein	0	+	YES
Os.55011.1.S1_x_at	LOC_Os01g20120	11427774	Expressed protein	0	-	YES
Os.45751.1.A1_x_at	LOC_Os01g20880	11637965	Protein kinase domain containing protein	1.00E-123	+	YES
Os.13500.2.S1_x_at	LOC_Os01g20940	11676292	Putative dual specificity protein phosphatase family protein	0	+	YES
Os.35123.1.S1_at	LOC_Os01g22230	12482404	Peroxidase family protein	0	+	YES
OsAffx.23355.1.S1_s_at	LOC_Os01g23630	13274139	Transcription initiation factor IID, 18kD subunit family protein	1.00E-104	+	NO
Os.24895.1.S1_at	LOC_Os01g24060	13543313	Putative Importin alpha-1b subunit protein	0	+	YES
Os.33510.1.S2_at	LOC_Os01g25320	14285672	TolA protein	0	+	YES
Os.25255.1.S1_at	LOC_Os01g25530	14454283	Putative PPR986-12 protein	0	+	YES
Os.18293.1.S1_at	LOC_Os01g26020	14734782	Expressed protein	1.00E-44	+	YES
Os.40545.1.S1_x_at	LOC_Os01g26160	14792157	Putative HASTY protein	0	+	YES
Os.12845.1.S1_at	LOC_Os01g26832	15177467	Hypothetical protein	1.00E-178	+	YES
Os.4023.1.S1_at	LOC_Os01g27020	15386316	Putative transposon protein, unclassified	0	+	YES

^a ^Rice gene models recorded from rice pseudomolecules, release 4 of the Institute of Genomic Research (TIGR).

^b ^Putative proteins were annotated by BLASTN search of TIGR rice pseudomolecules, release 4.

^c ^FL478 allele exactly matches the sequence of IR29 allele (+) or does not (-)

### Validation of SFPs in *Saltol *region by amplicon sequencing

**Figure 5:**
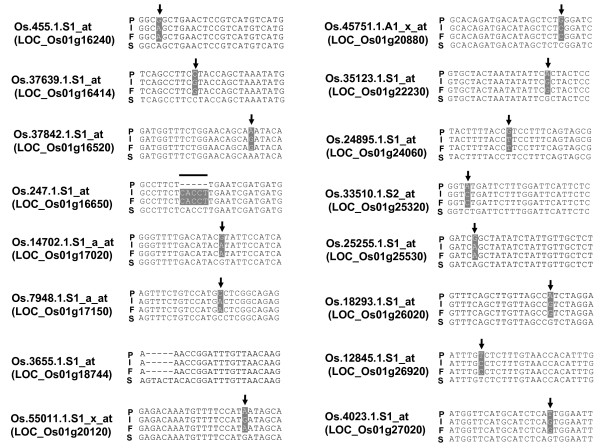
**Alignments of SFPs in the *Saltol *region**. Polymorphic residues are highlighted in gray. The locus corresponding to each probe set is indicated in parentheses. Arrows indicate SNPs. Bar, INDEL. P, Pokkali; I, IR29; F, FL478; S, target sequence from SIF.

**Figure 6:**
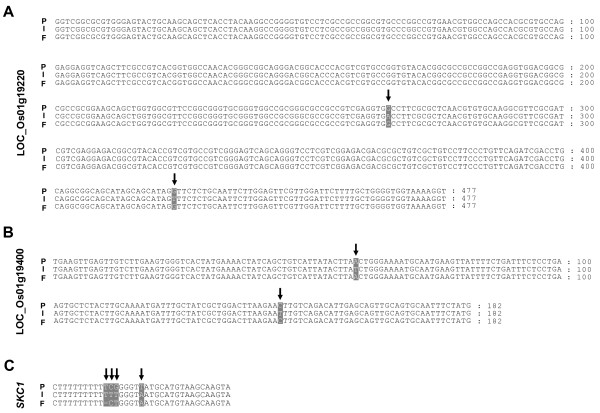
**Alignments of amplicon sequences of genes in a small segment of the *Saltol *region from the non-IR29 parent**. Polymorphic residues of (A) LOC_Os01g19220, (B) LOC_Os01g19400 and (C) LOC_Os01g20160 (*SKC1 *gene) are highlighted in gray. Arrows indicate SNPs or INDEL. P, Pokkali; I, IR29; F, FL478.

In order to confirm the SFPs detected in the *Saltol *region, we examined the SFP locations by amplicon sequencing. Alignments of the amplicon sequences are shown in Figure 5. For probe set Os.55011.1.S1_x_at, which corresponds to gene model LOC_Os01g20120, one SNP was found in the amplicon sequence at the SFP probe position and the FL478 allele was the same as in the Pokkali genotype. These results confirmed the SFP detection data, which suggested that FL478 contains a Pokkali-derived gene near *SKC1 *(LOC_Os01g20160). To further examine this region we checked additional genes in the vicinity of LOC_Os01g20120. We found that three additional genes (LOC_Os01g19220, LOC_Os01g19400, and LOC_Os01g20160 [*SKC1*]) within a < 1 Mb segment of FL478 also are of a non-IR29 origination (Figure 6). Taken together, it appears that FL478 contains a small non-IR29 haplotype block including *SKC1 *(Figure 4C), which we did not detect previously. We could not detect a SFP in the *SKC1 *gene in either the previous work or the present study because the expression level from the probe set (Os.30563.1.S1_at) for *SKC1 *was not "present" in all expression datasets, which is a requirement of our statistical filtering method. The *SKC1 *sequences are shown in Figure 6C. Surprisingly, in an apparently highly variable region, FL478 contains a haplotype that is not identical to either of the presumed parents. We confirmed this by sequencing amplicons from independent reactions from each genotype, making use of high fidelity Taq polymerase (Platinum *pfx *DNA polymerase, Invitrogen, USA). The existence in FL478 of an allele that matches neither IR29 nor the genotype which we know as Pokkali could be explained by either parent being genetically not uniform when the crosses to make RILs including FL478 were made. This notion is consistent with records now showing that there are actually at least eight distinct accessions named Pokkali in the germplasm collection at International Rice Research Institute .

### Correct SFP call rate by RPP method

We examined a total of 64 putative SFPs by amplicon sequencing (Additional file 2). Among them, 62 were found to cover polymorphisms (~97% validation). Among these 62 confirmed SFPs, 51 (82.2%) were positioned over a single SNP, seven (11.3%) were positioned over an INDEL, two (3.2%) spanned one SNP and one INDEL, one (1.6%) spanned > 1 SNP and no INDEL, and one spanned > 1 SNP and > 1 INDEL. From this we assert that at the threshold of top 20 percentile outlying scores, our detection method is correct about 97% of the time (2 false positive in 64) in a priori identification of SFPs from the Affymetrix rice genome array data using RNA-based datasets. Winzeler et al. (1998) identified more than 3,000 polymorphisms between two yeast strains at a 5% error rate using DNA hybridization [4]. Also, about 1,000 SFPs were identified at 3~7% error rates in yeast using mRNA hybridization [5]. In Arabidopsis, among 3,806 predicted SFPs, 97% of known polymorphisms were detected, which established a false negative rate of 3% [1]. Rostoks et al. (2005) used a probe level analysis of transcriptome data in barley to identify 10,504 putative SFPs, which included ~40% false positives [13]. More recently, rice genomic DNA was hybridized to an oligonucleotide microarray to detect SFPs [6] with an up to 20% false discovery rate. The 97% validation rate (3% false positives) from our method of RNA-based SFP detection by RPP compares favourably to these other performance metrics.

In the single nucleotide polymorphism database (dbSNP) of the National Center for Biotechnology Information (NCBI), more than 5 million polymorphisms including SNPs, small INDELs and microsatellite repeat variations have been catalogued. Also, the International Rice Research Institute has initiated a project to identify a large fraction of the SNPs in germplasm pertinent to cultivated rice through whole-genome comparisons [14]. This will provide additional millions of rice SNPs. Our work has shown that the existing Affymetrix rice genome array can be used to provide some thousands of SFP markers from a pairwise rice genotype comparison. Because a number of researchers have been using Affymetrix microarrays for transcriptome analyses in a range of rice RILs, NILs and germplasm accessions, existing data files provide abundant opportunities for the identification of additional SFP markers and resolution of trait determinants without additional expenditure on materials or data acquisition. Therefore, application of the RPP method to existing data could augment, or sometimes obviate the need for, other markers to meet objectives such as map-based cloning and sub-Mb resolution of the position of trait determinants. Examples of such applications would be to define introgressed regions in NILs or to generate moderate density linkage maps from RIL populations. Also, SFPs can provide a reliable discovery component in the development of markers for other detection systems including SNPs, CAPS, DArT, and SSRs.

## Conclusion

We identified a small (< 1 Mb) segment from the salt tolerant parent, presumably a Pokkali accession, in the *Saltol *region of RIL FL478 using SFP analysis with confirmation by amplicon sequencing. This small segment is flanked by alleles identical to those in the salt sensitive parent IR29. This study shows that the Affymetrix rice genome array, designed for expression analysis, provides a satisfactory genetic marker system for mapping in rice using RNA hybridization and the RPP method of SFP analysis.

## Methods

### Plant materials

Seeds of rice (*Oryza sativa*) genotypes Pokkali, IR29 and FL478 were obtained from G. B. Gregorio at the International Rice Research Institute in the Philippines and then propagated at the USDA/ARS George E. Brown, Jr., US Salinity Laboratory in Riverside, CA. Seedlings of the three genotypes were grown and stored at -80°C until DNA extraction.

### Genomic DNA isolation

Genomic DNA was extracted from seedlings of the three genotypes using a DNeasy Plant Mini Kit (Qiagen, USA) according to the manufacturer's protocol. For each genotype, more than seven seedlings were ground and about 0.1 g of pulverized tissue was processed. Purified genomic DNA was quantified at 260 nm using a spectrophotometer.

### SFP identification by RPP method

We produced RNA expression data using the Affymetrix rice GeneChip hybridized with cRNA synthesized from shoot tissue RNA of young seedling of three rice genotypes with and without salt stress, essentially as described previously [3]. The dataset was from seven chips with Pokkali RNA, five chips with IR29 and six chips with FL478. The Affymetrix rice GeneChip consists of probe sets designed for 48,564 *japonica *and 1,260 *indica *sequences . For SFP detection, we applied the RPP method to each probe set that had a "present" call in all chip samples from each pair of genotypes under comparison: (1) Pokkali versus IR29, (2) Pokkali versus FL478, (3) IR29 versus FL478. Using the top 20 percentile of all overall outlying scores as a cutoff, SFP probes were compiled. FL478 alleles presumed to be inherited from IR29 were then obtained as the SFPs detected in comparisons (1) and (2) but not (3). Similarly FL478 alleles presumed to be from Pokkali were obtained as the SFPs detected in (1) and (3) but not (2). As described in Cui et al. (2005) [2], the RPP method first measures the overall outlyingness of each probe set. Probe sets with significantly high outlying scores are then analyzed at the probe level and the probes that make a sufficiently large contribution to overall outlyingness of the probe set are identified as SFP probes.

### Primer design

We obtained the target sequence of each probe set from the sequence information file (SIF) for the Affymetrix rice genome array . The target sequence corresponds to the 5' end of the 5'-most probe to the 3' end of the 3'-most probe. To obtain the corresponding *indica *rice genomic sequences, each target sequence was searched using BLASTN against the *indica *rice whole genome shotgun sequences in the NCBI database . The *indica *sequences (cv. 93-11) were aligned with the target sequence using AlignX in Vector NTI Advance 10 (Invitrogen, USA). HarvEST:RiceChip [15] was used to check the position of SFP probes in each target sequence. Primers were designed using Primer3 [16]. The primers are listed in Additional file 3.

### PCR

PCR was performed in 20 μl containing 25~50 ng of genomic DNA, 0.1 μM of specific primers, 0.2 mM dNTPs, and 1 unit of Taq (GenScript Corp., USA) DNA polymerase. The reaction included a 5 min denaturation at 95°C followed by 35 cycles of PCR (94°C, 30 sec; 55~65°C, 70 sec; 72°C, 60 sec), and a final 5 min at 72°C. Aliquots (4 μl) of the PCR products were separated on a 1.2% agarose gel to check the band size and quantity. PCR products were purified using QIAquick PCR purification Kit (Qiagen, USA) to prepare for sequencing.

### DNA sequence analysis

DNA sequencing was performed by the dideoxynucleotide chain termination method [17]. The amplified PCR products (amplicons) were sequenced with an ABI-PRISM 3730×l Autosequencer (ABI, USA). These sequences were then compared with the target sequence of each probe set using AlignX (Invitrogen, USA). Comparisons of nucleotide sequence similarity were displayed using GeneDoc [18]. Rice genomic amplicon sequences have been deposited in the GenBank Data Library under accession numbers [GenBank:EF589163–EF589342 and EU099042–EU099056].

## Authors' contributions

SHK, HW, AMI and TJC designed the experiment. SHK, PRB, and HW performed the research. XC and JX accomplished the statistical analysis. SW produced HarvEST:RiceChip. CW provided the plant materials. SHK and TJC wrote most of the paper. All authors read and approved the final manuscript.

## Authors' information

Current address of JX is Department of Statistics and Actuarial Science, East China Normal University, Shanghai 200241, China. Current address of HW is Department of Plant Pathology, University of California, Davis, CA 95616, USA.

## Supplementary Material

Additional file 1 **SFP probe sets detected in this study, their probe numbers, predicted origin of each FL478 allele, and other information**. The data provided represent information about SFP probe sets including gene model, annotation, the probe numbers and predicted origin of each FL478 allele. Click here for file

Additional file 2 **Sequenced SFP probe sets and the information of each SFP position**. The data show the information including gene models, chromosome numbers of sequenced SFP probe sets, and nucleotide sequences at SNP or INDEL of each SFP position. Click here for file

Additional file 3 **Primer list and amplicon lengths of sequenced SFP-containing probe sets**. The data represent primer sequences for amplicon sequencing of the SFP-containing probe sets and their amplicon lengths. Click here for file
