# Supplementary material for: Detection and validation of single feature polymorphisms using RNA expression data from a rice genome array
Source: BMC Plant Biol. 2009 May 29;9:65. doi: 10.1186/1471-2229-9-65 (PMC2697985; doi:10.1186/1471-2229-9-65)
Supplement: Additional file 1 — SFP probe sets detected in this study, their probe numbers, predicted origin of each FL478 allele, and other information. The data provided represent information about SFP probe sets including gene model, annotation, the probe numbers and predicted origin of each FL478 allele. [file 1471-2229-9-65-S1.pdf]

Additional file 1

SFP probe sets detected in this study, their probe numbers, predicted origin of each FL478 allele, and other information

Probe set name	Gene model	Annotation	Probe #	FL478 allele from
Os.10094.1.S1_at	LOC_Os07g47580	TGF beta-inducible nuclear protein 1, putative, expressed	3	IR29
Os.10104.1.S1_at	LOC_Os04g40980	Uncharacterized BCR, COG1963 family protein, expressed	6	IR29
Os.10115.1.S1_at	LOC_Os01g36460	Myb-related protein Hv33, putative, expressed	9	IR29
Os.10123.1.S1_at	LOC_Os01g58114	expressed protein	3	IR29
Os.10134.1.S1_at	LOC_Os11g11390	60S ribosomal protein L10-1, putative, expressed	3	IR29
Os.10137.1.S1_a_at	LOC_Os06g07830	Clathrin assembly protein, putative, expressed	9	IR29
Os.10188.1.S1_at	LOC_Os08g27070	expressed protein	9	IR29
Os.10221.1.S1_at	LOC_Os01g47770	Nuclear movement protein, expressed	9	IR29
Os.10224.1.S1_at	LOC_Os02g40000	Sedlin, N-terminal conserved region family protein, expressed	8	IR29
Os.10287.1.S1_at	LOC_Os07g01370	Peroxidase 1 precursor, putative, expressed	4	IR29
Os.10353.1.S1_at	LOC_Os01g07400	SVP1-like protein 2, putative, expressed	7	IR29
Os.10370.2.S1_at	LOC_Os07g47640	Photosystem II reaction centre X protein containing protein, expressed	1	IR29
Os.10376.1.S1_at	LOC_Os02g34884	SacI homology domain containing protein, expressed	4	IR29
Os.10391.1.S1_a_at	LOC_Os05g51630	early-responsive to dehydration protein, putative, expressed	6	IR29
Os.10417.1.S1_at	LOC_Os08g40830	Pumilio-family RNA binding repeat containing protein, expressed	4	IR29
Os.10429.1.S1_at	LOC_Os08g03650	transposon protein, putative, Mutator sub-class, expressed	6	IR29
Os.10451.1.S1_at	LOC_Os07g31260	expressed protein	8	IR29
Os.10518.1.S1_at	LOC_Os09g34300	Inositol 1, 3, 4-trisphosphate 5/6-kinase family protein, expressed	4	IR29
Os.10603.1.S1_at	LOC_Os03g12590	Adaptin N terminal region family protein, expressed	7	IR29
Os.10662.1.S1_at	LOC_Os01g15320	Rapid ALkalinization Factor family protein, expressed	2	IR29
Os.10666.1.S1_at	LOC_Os01g15260	Dynein light chain 1, cytoplasmic, putative, expressed	8	IR29
Os.10719.1.S1_at	LOC_Os02g58460	Beta-catenin-like protein 1, putative, expressed	6	IR29
Os.10843.1.S1_at	LOC_Os02g49800	Eukaryotic aspartyl protease family protein, expressed	6	IR29
Os.10883.1.S1_at	LOC_Os08g28680	Ubiquitin-conjugating enzyme E2 M, putative, expressed	5	IR29
Os.10885.1.S1_at	LOC_Os09g20650	F-box protein interaction domain containing protein, expressed	6	IR29
Os.10985.1.S1_at	LOC_Os11g09280	Protein disulfide-isomerase precursor, putative, expressed	2	IR29
Os.11041.1.S1_s_at	LOC_Os11g26890	expressed protein	0	IR29
Os.11140.1.S1_at	LOC_Os03g49170	Zinc finger C-x8-C-x5-C-x3-H type family protein, expressed	6	IR29
Os.11143.1.S1_at	LOC_Os03g51980	expressed protein	0	IR29
Os.11216.1.S1_at	LOC_Os03g55090	glycogen/starch/alpha-glucan phosphorylases family protein, expressed	3	IR29
Os.11264.1.S1_at	LOC_Os03g15630	Harpin-induced protein 1 containing protein, expressed	4	IR29
Os.11265.1.S1_at	LOC_Os03g16670	haloacid dehalogenase-like hydrolase family protein, putative, expressed	7	IR29
Os.11276.1.S1_at	LOC_Os03g22180	60S ribosomal protein L18, putative, expressed	10	IR29
Os.11297.1.S1_at	LOC_Os12g38000	60S ribosomal protein L2, putative, expressed	9	IR29
Os.11347.1.S1_at	LOC_Os01g59120	G2/mitotic-specific cyclin S13-6, putative, expressed	1	IR29
Os.11380.1.S1_x_at	LOC_Os12g35590	expressed protein	9	IR29
Os.11416.1.S1_at	LOC_Os11g43610	oxidoreductase, 2OG-Fe oxygenase family protein, putative, expressed	7	IR29
Os.11485.1.S1_at	LOC_Os01g66180	Cytochrome c, putative, expressed	8	IR29

Os.116.1.S1_at	LOC_Os01g15610	hypothetical protein	10	IR29
Os.11652.1.S1_at	LOC_Os08g15230	heat shock protein, putative, expressed	5	IR29
Os.11689.1.S1_at	LOC_Os01g34330	BAH domain containing protein, expressed	8	IR29
Os.11696.1.S1_at	LOC_Os01g70270	Auxin response factor 2, putative, expressed	1	IR29
Os.11699.1.S1_at	LOC_Os06g47320	T-complex protein 1, eta subunit, putative, expressed	4	IR29
Os.11718.1.S1_at	LOC_Os12g02980	GDA1/CD39 family protein, expressed	0	IR29
Os.11767.1.S1_at	LOC_Os04g47240	YDA, putative, expressed	0	IR29
Os.11790.1.S1_at	LOC_Os08g42560	Glycyl-tRNA synthetase, putative, expressed	6	IR29
Os.11898.1.S1_at	LOC_Os07g46160	speckle-type POZ protein, putative, expressed	10	IR29
Os.11919.1.S1_at	LOC_Os01g70790	XYPPX repeat family protein, expressed	1	IR29
Os.11937.1.S1_at	LOC_Os11g29370	haloacid dehalogenase-like hydrolase family protein, putative, expressed	7	IR29
Os.11944.1.S2_s_at	LOC_Os09g20350	AP2 domain containing protein, expressed	6	IR29
Os.11960.1.S1_at	LOC_Os07g48160	Alpha-galactosidase precursor, putative, expressed	4	IR29
Os.11975.2.A1_at	LOC_Os01g68300	expressed protein	8	IR29
Os.11981.1.S1_x_at	LOC_Os02g39730	expressed protein	8	IR29
Os.12064.1.S1_at	LOC_Os12g05080	expressed protein	7	IR29
Os.12079.1.S1_at	LOC_Os04g40860	transposon protein, putative, unclassified, expressed	10	IR29
Os.12088.1.S1_at	LOC_Os06g14550	Signal recognition particle 9 kDa protein, putative, expressed	8	IR29
Os.12102.1.S1_at	LOC_Os05g07632	haloacid dehalogenase-like hydrolase family protein, putative, expressed	10	IR29
Os.12188.1.S1_at	LOC_Os02g10200	AN1-like Zinc finger family protein, expressed	6	IR29
Os.12211.1.S1_at	LOC_Os08g15170	ATP synthase epsilon chain, mitochondrial, putative, expressed	2	IR29
Os.12291.1.S1_at	LOC_Os06g48780	60S acidic ribosomal protein P3, putative, expressed	0	IR29
Os.12298.1.S1_at	LOC_Os06g14530	expressed protein	5	IR29
Os.12311.1.S1_x_at	LOC_Os01g01450	stress responsive protein, putative, expressed	9	IR29
Os.12314.1.S1_at	LOC_Os05g07650	Endomembrane protein 70 containing protein, expressed	1	IR29
Os.12342.1.S2_at	LOC_Os11g04420	Uncharacterised protein family containing protein, expressed	9	IR29
Os.12347.3.S1_at	LOC_Os12g04180	RNA recognition motif family protein, expressed	1	IR29
Os.12362.1.S1_at	LOC_Os03g37970	60S ribosomal protein L13-2, putative, expressed	10	IR29
Os.12378.1.S1_at	LOC_Os07g36480	vacuolar ATP synthase subunit H 2, putative, expressed	4	IR29
Os.12409.1.S1_at	LOC_Os07g02340	expressed protein	10	IR29
Os.12427.1.S1_a_at	LOC_Os07g47194	GEX1, putative, expressed	4	IR29
Os.12427.2.S1_x_at	LOC_Os07g47194	GEX1, putative, expressed	9	IR29
Os.12448.1.S1_at	LOC_Os06g06870	MIZ zinc finger family protein, expressed	9	IR29
Os.12491.1.S1_at	LOC_Os06g17870	nitrate-induced NOI protein, putative	5	IR29
Os.12579.1.S1_at	LOC_Os09g07830	Acetyl-CoA acetyltransferase, cytosolic 1, putative, expressed	8	IR29
Os.12665.1.S1_at	LOC_Os07g06740	Calcium-dependent protein kinase, isoform AK1, putative, expressed	2	IR29
Os.12681.1.A1_at	LOC_Os03g43760	protein kinase, putative, expressed	9	IR29
Os.12702.1.S1_at	LOC_Os12g21890	PTEN, putative, expressed	2	IR29
Os.12799.1.S1_s_at	LOC_Os05g23850	expressed protein	10	IR29
Os.12806.1.S2_at	LOC_Os03g28310	DnaJ domain containing protein, expressed	10	IR29
Os.12839.1.S1_at	LOC_Os04g40730	oxidoreductase, short chain dehydrogenase/reductase family protein, expressed	8	IR29

Os.12845.1.S1_at	LOC_Os01g26832	expressed protein	10	IR29
Os.12933.1.S1_at	LOC_Os04g58640	nudix type motif 21, putative, expressed	10	IR29
Os.12974.1.S1_at	LOC_Os11g29380	DNA replication licensing factor MCM2, putative, expressed	6	IR29
Os.13500.2.S1_x_at	LOC_Os01g20940	dual specificity protein phosphatase family protein, putative, expressed	3	IR29
Os.13560.1.S1_at	LOC_Os01g27790	retrotransposon protein, putative, unclassified	9	IR29
Os.13862.1.S1_at	LOC_Os09g30466	RNase Z, putative, expressed	1	IR29
Os.13973.2.S1_s_at	LOC_Os05g29020	expressed protein	8	IR29
Os.13973.3.S1_at	LOC_Os05g29030	protein phosphatase 2C, putative, expressed	2	IR29
Os.14071.1.S1_at	LOC_Os09g29940	Auxin-independent growth promoter, putative, expressed	8	IR29
Os.14080.1.S1_s_at	LOC_Os03g47120	CBS domain-containing protein, putative, expressed	3	IR29
Os.14090.1.S1_a_at	LOC_Os01g67590	Peptidase family M3 containing protein, expressed	9	IR29
Os.14099.1.S1_a_at	LOC_Os02g02350	Kelch motif family protein, expressed	4	IR29
Os.14157.1.S1_at	LOC_Os08g15500	expressed protein	4	IR29
Os.14167.1.S1_at	LOC_Os06g14740	expressed protein	10	IR29
Os.14215.1.S1_at	LOC_Os04g40370	F-box domain containing protein, expressed	4	IR29
Os.14496.1.S1_at	LOC_Os08g09270	pentatricopeptide, putative, expressed	8	IR29
Os.14508.1.S1_at	LOC_Os03g60730	BED zinc finger family protein, expressed	10	IR29
Os.14555.1.S1_at	LOC_Os10g27990	exocyst complex component Sec15, putative, expressed	6	IR29
Os.14601.1.S1_at	LOC_Os03g16090	LIM domain-containing protein, putative, expressed	7	IR29
Os.14625.1.S1_at	LOC_Os01g14810	5' nucleotidase family protein, putative, expressed	10	IR29
Os.14702.1.S1_a_at	LOC_Os01g17020	expressed protein	7	IR29
Os.14719.1.S1_at	LOC_Os09g12770	transfactor, putative, expressed	9	IR29
Os.14760.1.S1_at	LOC_Os07g32430	Pre-mRNA splicing factor ATP-dependent RNA helicase PRP16, putative, expressed	9	IR29
Os.14762.1.S1_x_at	LOC_Os11g47920	GRAS family transcription factor containing protein	1	IR29
Os.14813.1.S1_at	LOC_Os11g42790	sodium/hydrogen exchanger 3 family protein, expressed	7	IR29
Os.14894.3.A1_a_at	LOC_Os03g15600	expressed protein	8	IR29
Os.14915.1.S1_a_at	LOC_Os12g37910	GDSL-like Lipase/Acylhydrolase family protein, expressed	6	IR29
Os.15107.1.S1_at	LOC_Os12g30540	ubiquitin-specific protease 12, putative, expressed	5	IR29
Os.15138.1.S1_at	LOC_Os01g41900	MCB2 protein, putative, expressed	4	IR29
Os.15221.1.S1_a_at	LOC_Os05g26914	Chaperone protein dnaJ, putative, expressed	9	IR29
Os.15236.1.S1_at	LOC_Os04g40850	26S proteasome non-ATPase regulatory subunit 6, putative, expressed	3	IR29
Os.15349.1.S1_a_at	LOC_Os06g03860	SPX domain-containing protein, putative, expressed	10	IR29
Os.15394.1.S1_s_at	LOC_Os01g31360	expressed protein	1	IR29
Os.15454.1.S2_x_at	LOC_Os06g06760	U-box domain containing protein, expressed	9	IR29
Os.15541.2.S1_x_at	LOC_Os01g01830	Prolyl endopeptidase, putative, expressed	5	IR29
Os.15564.1.S1_at	LOC_Os02g39840	Eukaryotic initiation factor, putative, expressed	3	IR29
Os.15587.2.A1_a_at	LOC_Os04g41100	protein kinase family protein, putative, expressed	9	IR29
Os.15593.1.S1_at	LOC_Os03g15740	calcium-binding EF hand family protein, putative, expressed	3	IR29
Os.15810.2.S1_x_at	LOC_Os06g39760	Serine-threonine kinase receptor-associated protein, putative, expressed	8	IR29
Os.15822.1.S1_s_at	LOC_Os08g32980	expressed protein	5	IR29
Os.15900.1.A1_at	LOC_Os06g03220	expressed protein	9	IR29

Os.15957.1.S1_at	LOC_Os06g11800	Annexin-like protein RJ4, putative, expressed	4	IR29
Os.15967.1.S1_at	LOC_Os02g54910	transport protein, putative, expressed	7	IR29
Os.16062.1.S1_at	LOC_Os10g22980	Leucine Rich Repeat family protein, expressed	4	IR29
Os.16183.1.S1_at	LOC_Os01g39134	EF hand family protein, expressed	4	IR29
Os.16333.1.S1_at	LOC_Os04g40310	isocitrate dehydrogenase, NAD-dependent family protein, expressed	3	IR29
Os.16334.1.S1_at	LOC_Os06g15370	POT family protein, expressed	4	IR29
Os.16394.1.S1_at	LOC_Os04g44840	Glycosyl hydrolase family 3 C terminal domain containing protein, expressed	10	IR29
Os.16410.1.S1_s_at	LOC_Os06g07820	BSD domain containing protein, expressed	5	IR29
Os.16914.1.S2_at	LOC_Os02g27400	F-box protein interaction domain containing protein, expressed	10	IR29
Os.17010.1.S1_at	LOC_Os09g29470	expressed protein	4	IR29
Os.17037.1.S2_a_at	LOC_Os03g15600	expressed protein	9	IR29
Os.17118.1.A1_at	LOC_Os01g07410	Axi 1 protein, putative, expressed	6	IR29
Os.17164.1.S1_s_at	LOC_Os02g32970	hydrolase, alpha/beta fold family protein, putative, expressed	10	IR29
Os.17200.1.S1_at	LOC_Os11g09310	Zinc knuckle family protein, expressed	5	IR29
Os.17282.1.S1_at	LOC_Os11g08670	ATP-NAD kinase family protein, expressed	10	IR29
Os.17305.1.A1_s_at	LOC_Os06g43840	Protein kinase domain containing protein, expressed	6	IR29
Os.17486.2.A1_at	LOC_Os05g07300	D-mannose binding lectin family protein, expressed	9	IR29
Os.17516.1.S1_at	LOC_Os04g40840	radical SAM domain-containing protein, putative, expressed	10	IR29
Os.17533.1.S1_at	LOC_Os11g08950	protein kinase family protein, putative, expressed	2	IR29
Os.17665.1.S1_x_at	LOC_Os06g14190	NF-X1 type zinc finger family protein, expressed	5	IR29
Os.17777.1.S1_x_at	LOC_Os01g33784	lipase family protein, putative, expressed	8	IR29
Os.17777.2.S1_x_at	LOC_Os01g33784	lipase family protein, putative, expressed	4	IR29
Os.17863.1.S1_at	LOC_Os06g12260	expressed protein	7	IR29
Os.17971.1.S1_at	LOC_Os04g02150	Eukaryotic initiation factor 3, gamma subunit family protein, expressed	5	IR29
Os.18001.2.S1_x_at	LOC_Os01g44040	O-acetyltransferase, putative, expressed	9	IR29
Os.18009.1.S1_a_at	LOC_Os07g46440	ribosomal protein S9 containing protein, expressed	3	IR29
Os.18016.1.S1_at	LOC_Os07g01990	expressed protein	0	IR29
Os.18123.1.S1_at	LOC_Os09g19900	transducin family protein, putative, expressed	9	IR29
Os.18149.1.S1_x_at	LOC_Os04g40740	Kelch motif family protein, expressed	9	IR29
Os.18215.1.S1_at	LOC_Os06g30310	Alpha-glucan water dikinase, chloroplast precursor, putative, expressed	1	IR29
Os.18293.1.S1_at	LOC_Os01g26020	expressed protein	5	IR29
Os.18300.1.S1_at	LOC_Os06g40210	expressed protein	7	IR29
Os.18305.1.S1_at	LOC_Os05g50550	Polyprenyl synthetase family protein, expressed	5	IR29
Os.18357.1.S1_at	LOC_Os07g32460	SH3 domain-containing protein 3, putative, expressed	1	IR29
Os.18419.1.S1_at	LOC_Os03g63450	SnRK1-interacting protein 1, putative, expressed	2	IR29
Os.18523.1.S1_at	LOC_Os12g07050	Ssu72-like protein, expressed	4	IR29
Os.18552.1.S1_at	LOC_Os11g31980	Serine carboxypeptidase family protein, expressed	10	IR29
Os.18599.1.S1_at	LOC_Os03g57970	Protease inhibitor/seed storage/LTP family protein, expressed	10	IR29
Os.18612.1.S1_x_at	LOC_Os04g36050	transmembrane CLPTM1 family protein, putative, expressed	5	IR29
Os.18627.1.S1_at	LOC_Os03g53950	Strictosidine synthase family protein, expressed	6	IR29
Os.18630.1.S1_at	LOC_Os11g26190	expressed protein	10	IR29

Os.187.1.S1_at	LOC_Os09g32680	G1/S-specific cyclin C-type, putative, expressed	7	IR29
Os.18714.1.S2_at	LOC_Os10g14920	nodulin MtN21 family protein, putative, expressed	7	IR29
Os.18716.1.S1_at	LOC_Os04g42320	AT hook motif family protein, expressed	6	IR29
Os.18721.1.S1_at	LOC_Os02g10230	ZIP Zinc transporter family protein, expressed	1	IR29
Os.18761.1.S1_at	LOC_Os04g36620	hydrolase, alpha/beta fold family protein, expressed	4	IR29
Os.18869.1.S1_at	LOC_Os06g50880	transducin family protein, putative, expressed	8	IR29
Os.18880.1.S1_at	LOC_Os11g23170	Mitochondrial carrier protein, expressed	9	IR29
Os.19040.1.S1_at	LOC_Os04g39220	expressed protein	6	IR29
Os.19079.1.S1_at	LOC_Os01g69130	Dynamin-related protein 3A, putative, expressed	2	IR29
Os.19107.1.S1_s_at	LOC_Os05g08850	Cytochrome P450 family protein, expressed	7	IR29
Os.19344.1.S1_at	LOC_Os01g41220	expressed protein	6	IR29
Os.19371.1.S1_at	LOC_Os12g29580	protein kinase, putative, expressed	8	IR29
Os.19421.1.S1_at	LOC_Os02g58570	PRLI-interacting factor A, putative, expressed	2	IR29
Os.1955.1.S1_at	LOC_Os07g02350	Casein kinase II, alpha chain, putative, expressed	2	IR29
Os.19566.1.S1_at	LOC_Os03g27820	expressed protein	3	IR29
Os.19570.1.S1_at	LOC_Os06g06880	Serine/threonine protein phosphatase PP1, putative, expressed	5	IR29
Os.19618.1.S1_at	LOC_Os01g39260	ATP-dependent metalloprotease FtsH family protein, expressed	9	IR29
Os.197.1.S1_a_at	LOC_Os01g15310	U6 snRNA-associated Sm-like protein LSm4, putative, expressed	7	IR29
Os.19942.1.S1_at	LOC_Os08g14620	expressed protein	10	IR29
Os.19983.2.S1_at	LOC_Os02g05440	expressed protein	9	IR29
Os.20214.1.S1_at	LOC_Os02g23826	expressed protein	6	IR29
Os.20288.1.S1_at	LOC_Os03g26960	expressed protein	8	IR29
Os.20312.1.S1_at	LOC_Os08g03310	Zinc finger C-x8-C-x5-C-x3-H type family protein, expressed	3	IR29
Os.20320.1.S1_at	LOC_Os11g09329	VHS domain containing protein, expressed	10	IR29
Os.20425.2.S1_x_at	LOC_Os12g25700	UDP-glucose 6-dehydrogenase, putative, expressed	10	IR29
Os.20575.1.S1_at	LOC_Os03g62240	expressed protein	10	IR29
Os.20709.1.S1_at	LOC_Os04g43140	DEAD/DEAH box helicase, putative, expressed	10	IR29
Os.20730.1.S1_at	LOC_Os09g34130	C2 domain-containing protein, putative, expressed	6	IR29
Os.20773.1.S1_at	LOC_Os06g10280	CDP-alcohol phosphatidyltransferase family protein, expressed	2	IR29
Os.21061.1.S1_at	LOC_Os07g47110	Tubby protein, putative, expressed	10	IR29
Os.21245.1.S1_at	LOC_Os11g11790	expressed protein	6	IR29
Os.21343.1.S1_at	LOC_Os05g11850	glycoside hydrolase family 47 protein, putative, expressed	8	IR29
Os.21399.1.S1_a_at	LOC_Os07g06980	histone deacetylase HDA110 isoform 1, putative, expressed	2	IR29
Os.21763.1.S1_at	LOC_Os03g38000	40S ribosomal protein S3, putative, expressed	10	IR29
Os.21786.1.S1_at	LOC_Os03g01190	oxidoreductase, zinc-binding dehydrogenase family protein, expressed	8	IR29
Os.2225.1.S1_at	LOC_Os04g44870	Elicitor-responsive protein 3, putative, expressed	8	IR29
Os.22373.1.S1_at	LOC_Os01g12860	Myb-like DNA-binding domain containing protein, expressed	6	IR29
Os.22452.1.S1_at	LOC_Os12g30030	HesB/YadR/YfhF family protein, putative, expressed	3	IR29
Os.22665.1.S1_at	LOC_Os03g01890	rolled leaf1, putative, expressed	4	IR29
Os.22672.1.S1_at	LOC_Os06g04470	expressed protein	0	IR29
Os.22696.1.S1_at	LOC_Os11g06750	60S ribosomal protein L3, putative, expressed	6	IR29

Os.22793.1.S1_at	LOC_Os11g07940	expressed protein	3	IR29
Os.22967.1.S1_s_at	LOC_Os03g02070	heavy-metal-associated domain-containing protein, putative, expressed	6	IR29
Os.2307.1.S1_at	LOC_Os05g50360	Anaphase promoting complex subunit 10, putative, expressed	9	IR29
Os.23191.1.S1_at	LOC_Os09g20660	Brain protein 44, putative, expressed	2	IR29
Os.23313.3.S1_at	LOC_Os10g19880	large secreted protein, putative, expressed	9	IR29
Os.23567.1.S1_at	LOC_Os08g10649	GTP-binding protein ERG, putative, expressed	7	IR29
Os.23620.1.A1_a_at	LOC_Os01g12840	expressed protein	9	IR29
Os.23780.1.A1_at	LOC_Os04g41200	Lipase family protein, expressed	6	IR29
Os.23822.1.A1_at	LOC_Os03g27120	ICE-like protease p20 domain containing protein, expressed	9	IR29
Os.23840.1.S1_at	LOC_Os09g33960	expressed protein	10	IR29
Os.23925.1.S1_at	LOC_Os02g01180	Kinesin motor domain containing protein, expressed	9	IR29
Os.24021.1.S1_x_at	LOC_Os03g02080	expressed protein	8	IR29
Os.24059.1.A1_at	LOC_Os04g10380	glycine-rich protein, putative, expressed	1	IR29
Os.24073.1.S1_at	LOC_Os11g07030	expressed protein	1	IR29
Os.24154.1.A1_at	LOC_Os11g02640	expressed protein	1	IR29
Os.24356.1.A1_at	LOC_Os12g35630	Elongation factor TS family protein, expressed	8	IR29
Os.24360.1.S2_at	LOC_Os03g51270	F-box domain containing protein, expressed	8	IR29
Os.247.1.S1_at	LOC_Os01g16650	Ubiquitin-conjugating enzyme X, putative, expressed	8	IR29
Os.24765.1.A1_at	LOC_Os04g09530	pentatricopeptide, putative, expressed	6	IR29
Os.24828.1.S1_at	LOC_Os11g31620	F-box domain containing protein, expressed	6	IR29
Os.24895.1.S1_at	LOC_Os01g24060	Importin alpha-1b subunit, putative, expressed	9	IR29
Os.24955.1.A1_x_at	LOC_Os01g67200	ribosomal protein L13 family protein, putative	9	IR29
Os.25108.1.S1_at	LOC_Os11g11070	mRNA capping enzyme, C-terminal domain containing protein, expressed	10	IR29
Os.25255.1.S1_at	LOC_Os01g25530	PPR986-12, putative, expressed	1	IR29
Os.25396.1.S1_at	LOC_Os03g15800	potassium channel tetramerisation domain-containing protein, putative, expressed	3	IR29
Os.25491.1.S1_at	LOC_Os11g35090	Centromeric protein E, putative, expressed	3	IR29
Os.25518.1.S1_at	LOC_Os03g53530	WD-repeat protein 4, putative, expressed	1	IR29
Os.25521.2.S1_at	LOC_Os10g20510	expressed protein	9	IR29
Os.25560.1.S1_at	LOC_Os02g43130	kinesin heavy chain, putative, expressed	8	IR29
Os.25589.1.S1_at	LOC_Os11g25780	PB1 domain containing protein, expressed	10	IR29
Os.26049.1.S1_at	LOC_Os08g28980	RNA exonuclease 4, putative, expressed	7	IR29
Os.2613.1.S1_at	LOC_Os12g10720	Glutathione S-transferase, putative, expressed	2	IR29
Os.26354.2.S1_x_at	LOC_Os07g31540	Ubiquitin family protein, expressed	5	IR29
Os.26377.1.S1_at	LOC_Os01g60860	U-box domain containing protein, expressed	4	IR29
Os.26461.1.S1_at	LOC_Os06g48760	UDP-sugar pyrophospharylase, putative, expressed	7	IR29
Os.26472.1.S1_at	LOC_Os04g40630	TAZ zinc finger family protein, expressed	1	IR29
Os.26476.1.S1_at	LOC_Os03g02440	expressed protein	6	IR29
Os.26551.1.S1_at	LOC_Os02g13130	KH domain containing protein, expressed	6	IR29
Os.26733.2.S1_x_at	LOC_Os10g29274	expressed protein	2	IR29
Os.26733.3.S1_x_at	LOC_Os10g29274	expressed protein	2	IR29
Os.26742.1.S1_at	LOC_Os11g02660	hydrolase, alpha/beta fold family protein, expressed	3	IR29

Os.26764.1.A1_at	LOC_Os12g32630	F-box domain containing protein, expressed	9	IR29
Os.2678.1.S1_at	LOC_Os02g13870	Aquaporin NIP1.2, putative, expressed	10	IR29
Os.26798.1.S1_at	LOC_Os12g35630	Elongation factor TS family protein, expressed	8	IR29
Os.26923.1.A1_at	LOC_Os07g29820	NB-ARC domain containing protein, expressed	8	IR29
Os.26952.1.S1_at	LOC_Os01g66500	phosphoribosylformylglycinamidine synthase, chloroplast precursor, putative, expressed	3	IR29
Os.26987.1.A1_at	LOC_Os03g27950	Ser/Thr protein phosphatase family protein, expressed	8	IR29
Os.26998.1.S2_at	LOC_Os02g05660	ATP-dependent helicase DDX41, putative, expressed	6	IR29
Os.27016.2.S1_at	LOC_Os01g39810	mannosyltransferase, putative, expressed	4	IR29
Os.27024.1.S1_at	LOC_Os02g17360	pentatricopeptide, putative, expressed	8	IR29
Os.27032.1.S2_at	LOC_Os05g03574	expressed protein	9	IR29
Os.27059.1.S1_at	LOC_Os08g10070	Protein kinase domain containing protein, expressed	3	IR29
Os.27075.1.S1_at	LOC_Os05g25780	rhodanese-like domain-containing protein, putative, expressed	10	IR29
Os.27119.1.S1_at	LOC_Os02g55120	Translation initiation factor IF-3, C-terminal domain containing protein, expressed	2	IR29
Os.27136.1.S1_at	LOC_Os04g39660	F-box domain containing protein, expressed	8	IR29
Os.27139.1.A1_at	LOC_Os02g39910	K+ channel tetramerisation domain containing protein, expressed	9	IR29
Os.27158.1.S1_at	LOC_Os02g15594	protein phosphatase 2C, putative, expressed	9	IR29
Os.27183.1.S1_at	LOC_Os06g07100	Zinc finger, C3HC4 type family protein, expressed	7	IR29
Os.27186.1.S1_at	LOC_Os11g29840	expressed protein	6	IR29
Os.27196.1.S1_x_at	LOC_Os04g43220	Zinc finger, C3HC4 type family protein, expressed	8	IR29
Os.27207.1.S1_at	LOC_Os12g25200	Chloride channel protein CLC-a, putative, expressed	9	IR29
Os.27217.1.A1_at	LOC_Os11g39640	Zinc finger, C3HC4 type family protein, expressed	0	IR29
Os.27239.1.S1_at	LOC_Os01g48830	expressed protein	2	IR29
Os.27299.1.A1_at	LOC_Os01g04800	B3 DNA binding domain containing protein, expressed	8	IR29
Os.27334.1.S1_at	LOC_Os02g17350	VHS domain containing protein, expressed	6	IR29
Os.27354.1.S1_at	LOC_Os09g17600	expressed protein	8	IR29
Os.27377.1.S1_at	LOC_Os06g11040	expressed protein	2	IR29
Os.27388.1.S1_s_at	LOC_Os10g01470	Homeobox-leucine zipper protein HAT22, putative, expressed	4	IR29
Os.27428.1.S1_at	LOC_Os06g47300	RNA-binding protein, putative, expressed	4	IR29
Os.27452.1.S1_at	LOC_Os08g02860	transposon protein, putative, unclassified, expressed	6	IR29
Os.27455.1.S1_x_at	LOC_Os01g29820	expressed protein	7	IR29
Os.27480.1.S1_at	LOC_Os06g13650	Glycosyl hydrolases family 38 protein, expressed	8	IR29
Os.27569.3.S1_at	LOC_Os12g02690	expressed protein	10	IR29
Os.27637.1.S1_at	LOC_Os01g01050	R3H domain containing protein, expressed	8	IR29
Os.27717.1.S1_a_at	LOC_Os01g58470	expressed protein	4	IR29
Os.27741.1.S1_at	LOC_Os04g38700	Molybdenum cofactor biosynthesis protein C, putative, expressed	2	IR29
Os.27745.1.A1_at	LOC_Os06g12600	Fructokinase, putative, expressed	6	IR29
Os.27830.1.S1_at	LOC_Os07g01480	Oxygen evolving enhancer protein 3 containing protein, expressed	8	IR29
Os.27849.1.S1_at	LOC_Os08g07830	expressed protein	6	IR29
Os.27862.1.S1_at	LOC_Os11g38260	harpin binding protein 1, putative, expressed	0	IR29
Os.27983.1.S2_at	LOC_Os02g13850	Leucine Rich Repeat family protein, expressed	5	IR29
Os.28031.1.S1_at	LOC_Os06g45610	expressed protein	8	IR29

Os.28066.1.S2_at	LOC_Os02g01170	HECT-domain family protein, expressed	8	IR29
Os.28207.1.S1_at	LOC_Os01g34200	rRNA processing protein, putative, expressed	5	IR29
Os.28300.4.S1_x_at	LOC_Os03g22350	Brix domain containing protein, expressed	4	IR29
Os.28431.1.S1_at	LOC_Os06g14412	expressed protein	8	IR29
Os.28457.1.S1_at	LOC_Os11g48070	GDSL-like Lipase/Acylhydrolase family protein, expressed	10	IR29
Os.28462.1.S1_s_at	LOC_Os12g02290	Nonspecific lipid-transfer protein 5 precursor, putative, expressed	5	IR29
Os.2906.1.S1_at	LOC_Os03g54980	UPF0139 protein, putative, expressed	9	IR29
Os.29809.2.S1_x_at	LOC_Os01g18280	SNF7 family protein, expressed	4	IR29
Os.30044.1.S1_a_at	LOC_Os01g01870	Helix-loop-helix DNA-binding domain containing protein, expressed	8	IR29
Os.30361.2.S1_x_at	LOC_Os01g43360	Myosin tail family protein, expressed	1	IR29
Os.30485.1.S1_at	LOC_Os07g02250	expressed protein	4	IR29
Os.32193.2.S1_x_at	LOC_Os01g37120	transducin family protein, putative, expressed	10	IR29
Os.3228.1.S1_a_at	LOC_Os03g50090	transposon protein, putative, CACTA, En/Spm sub-class, expressed	9	IR29
Os.32450.1.S1_at	LOC_Os02g06370	DNA-binding protein, putative, expressed	9	IR29
Os.32504.1.S1_at	LOC_Os07g02100	Flavin-binding monooxygenase-like family protein, expressed	0	IR29
Os.32545.1.S1_at	LOC_Os05g49990	expressed protein	8	IR29
Os.32618.1.S1_at	LOC_Os06g02560	growth-regulating factor, putative, expressed	5	IR29
Os.33183.1.S1_at	LOC_Os01g01030	Monocopper oxidase precursor, putative, expressed	4	IR29
Os.33212.1.S1_at	LOC_Os01g67580	ABC transporter family protein, expressed	10	IR29
Os.33311.2.S1_at	LOC_Os03g02090	expressed protein	8	IR29
Os.33351.1.S1_at	LOC_Os02g32230	retrotransposon protein, putative, unclassified, expressed	10	IR29
Os.33510.1.S2_at	LOC_Os01g25320	TolA protein, expressed	4	IR29
Os.33553.2.S1_x_at	LOC_Os01g49760	expressed protein	1	IR29
Os.33665.1.S1_at	LOC_Os11g43510	Lipase family protein, expressed	10	IR29
Os.33723.1.S1_at	LOC_Os01g49950	transposon protein, putative, Mutator sub-class	0	IR29
Os.33732.3.S1_x_at	LOC_Os07g01770	C2 domain-containing protein, putative, expressed	6	IR29
Os.33852.1.S1_at	LOC_Os01g36630	jmjC domain containing protein, expressed	2	IR29
Os.33971.1.S1_at	LOC_Os01g12180	expressed protein	2	IR29
Os.34142.1.S1_at	LOC_Os01g07360	CENP-E like kinetochore protein, putative, expressed	3	IR29
Os.34400.1.S1_at	LOC_Os01g15770	expressed protein	10	IR29
Os.3448.1.S1_a_at	LOC_Os01g70300	aspartate kinase family protein, expressed	7	IR29
Os.3448.2.S1_x_at	LOC_Os01g70300	aspartate kinase family protein, expressed	10	IR29
Os.34523.1.S1_at	LOC_Os11g30500	HVA22-like protein e, putative, expressed	8	IR29
Os.34674.1.S1_at	LOC_Os01g01360	POT family protein, expressed	8	IR29
Os.35068.1.S1_at	LOC_Os12g18770	Oxysterol-binding protein, expressed	8	IR29
Os.35089.1.S1_at	LOC_Os04g40870	expressed protein	8	IR29
Os.35123.1.S1_at	LOC_Os01g22230	Peroxidase family protein, expressed	10	IR29
Os.35186.1.S1_s_at	LOC_Os03g22400	Ulp1 protease family, C-terminal catalytic domain containing protein, expressed	10	IR29
Os.35219.1.S1_a_at	LOC_Os02g37030	expressed protein	2	IR29
Os.35387.1.S1_at	LOC_Os08g27030	Lipid phosphate phosphatase 2, putative, expressed	0	IR29
Os.3545.1.S1_at	LOC_Os06g07960	Cystathionine beta-lyase, chloroplast precursor, putative, expressed	6	IR29

Os.35495.1.S1_s_at	LOC_Os01g16030	ADP-ribosylation factor, putative, expressed	1	IR29
Os.35502.1.A1_at	LOC_Os09g19830	YDG/SRA domain containing protein, expressed	9	IR29
Os.35785.1.S1_x_at	LOC_Os01g35160	receptor-like protein kinase 4, putative, expressed	3	IR29
Os.35800.1.S1_s_at	LOC_Os11g06040	phox domain-containing protein, putative, expressed	8	IR29
Os.35858.2.S1_x_at	LOC_Os01g44110	Protein kinase domain containing protein, expressed	0	IR29
Os.35889.1.S1_at	LOC_Os01g68550	pentatricopeptide, putative, expressed	2	IR29
Os.36403.1.S1_at	LOC_Os09g25050	pentatricopeptide, putative, expressed	4	IR29
Os.3655.1.S1_at	LOC_Os01g18744	Transferase family protein, expressed	10	IR29
Os.3697.1.S1_at	LOC_Os06g02210	Bark storage protein A precursor, putative, expressed	7	IR29
Os.36979.1.S1_x_at	LOC_Os09g33630	Protein kinase domain containing protein, expressed	0	IR29
Os.37011.1.S1_at	LOC_Os01g12800	Mpv17/PMP22 family protein, expressed	8	IR29
Os.37320.2.S1_x_at	LOC_Os03g62950	expressed protein	8	IR29
Os.37338.1.S1_at	LOC_Os07g01530	expressed protein	0	IR29
Os.3748.1.S1_at	LOC_Os01g12260	Vacuolar ATP synthase subunit E, putative, expressed	4	IR29
Os.3748.1.S1_at	LOC_Os01g12260	Vacuolar ATP synthase subunit E, putative, expressed	5	IR29
Os.37509.2.S1_x_at	LOC_Os01g50040	expressed protein	5	IR29
Os.37639.1.S1_at	LOC_Os01g16414	Actin family protein, expressed	9	IR29
Os.37737.1.S1_at	LOC_Os12g03090	40S ribosomal protein S16, putative, expressed	7	IR29
Os.37797.1.A1_s_at	LOC_Os02g31960	ATP/GTP binding protein, putative, expressed	10	IR29
Os.37842.1.S1_at	LOC_Os01g16520	glutamyl-tRNA synthetase family protein, expressed	6	IR29
Os.37966.1.S1_a_at	LOC_Os01g14690	expressed protein	7	IR29
Os.38074.1.S1_x_at	LOC_Os01g14790	expressed protein	10	IR29
Os.38168.1.S1_at	LOC_Os10g20600	Zinc finger, C3HC4 type family protein, expressed	0	IR29
Os.38168.2.S1_x_at	LOC_Os10g20600	Zinc finger, C3HC4 type family protein, expressed	1	IR29
Os.38233.1.S1_s_at	LOC_Os04g42600	Polyadenylate-binding protein 2, putative, expressed	2	IR29
Os.38249.1.S1_at	LOC_Os01g38530	early flowering 3, putative, expressed	8	IR29
Os.38404.1.S1_s_at	LOC_Os11g07870	Type III restriction enzyme, res subunit family protein, expressed	8	IR29
Os.38485.1.S1_at	LOC_Os03g26910	trehalose-phosphatase family protein, expressed	3	IR29
Os.38748.1.S1_s_at	LOC_Os11g42350	glutathione synthetase family protein, expressed	7	IR29
Os.38792.1.S1_at	LOC_Os06g47310	uncharacterized plant-specific domain TIGR01627 family protein	4	IR29
Os.38989.1.S1_at	LOC_Os06g48530	C2H2 zinc-finger protein, putative, expressed	10	IR29
Os.39054.1.A1_s_at	LOC_Os07g08840	Thioredoxin H-type 2, putative, expressed	10	IR29
Os.39472.1.A1_at	LOC_Os02g37050	Phosphatidylinositolglycan class N family protein, expressed	6	IR29
Os.39913.1.A1_at	LOC_Os07g07070	C2 domain containing protein, expressed	6	IR29
Os.39971.1.A1_at	LOC_Os02g03790	expressed protein	0	IR29
Os.39978.1.S1_at	LOC_Os08g14330	Possible apospory-associated protein C, putative, expressed	4	IR29
Os.40059.1.S1_at	LOC_Os02g58470	expressed protein	7	IR29
Os.40187.1.S1_at	LOC_Os01g03750	expressed protein	1	IR29
Os.4023.1.S1_at	LOC_Os01g27020	transposon protein, putative, unclassified, expressed	10	IR29
Os.40545.1.S1_x_at	LOC_Os01g26160	HASTY, putative, expressed	2	IR29
Os.41226.1.S1_at	LOC_Os01g43380	glycosyltransferase family protein 1, putative, expressed	6	IR29

Os.41638.1.S1_at	LOC_Os01g50060	expressed protein	6	IR29
Os.4208.1.S1_at	LOC_Os06g07070	Protein kinase domain containing protein, expressed	3	IR29
Os.4210.1.S1_at	LOC_Os06g07090	Adaptin N terminal region family protein, expressed	1	IR29
Os.43805.1.S1_x_at	LOC_Os01g14820	expressed protein	2	IR29
Os.43951.1.S1_at	LOC_Os03g16150	mannose-1-phosphate guanyltransferase, putative, expressed	9	IR29
Os.4407.1.S1_at	LOC_Os06g01980	Cell division control protein 48 homolog C, putative, expressed	9	IR29
Os.4417.1.S1_at	LOC_Os09g12290	Bifunctional aspartokinase/homoserine dehydrogenase 2, chloroplast precursor, putative,	7	IR29
Os.44821.1.A1_x_at	LOC_Os06g02620	DnaJ C terminal region family protein, expressed	6	IR29
Os.44823.1.S1_x_at	LOC_Os06g02550	Serine/threonine-protein kinase MAK, putative, expressed	2	IR29
Os.4506.3.S1_x_at	LOC_Os06g02370	expressed protein	0	IR29
Os.4513.1.S1_at	LOC_Os06g02440	expressed protein	0	IR29
Os.455.1.S1_at	LOC_Os01g16240	Calmodulin, putative, expressed	4	IR29
Os.45751.1.A1_x_at	LOC_Os01g20880	Protein kinase domain containing protein	3	IR29
Os.45994.1.S1_x_at	LOC_Os01g15790	expressed protein	6	IR29
Os.46002.1.S1_at	LOC_Os01g49490	expressed protein	8	IR29
Os.46044.1.S1_s_at	LOC_Os01g15020	WD-40 repeat family protein, putative, expressed	4	IR29
Os.46046.1.A1_at	LOC_Os07g36490	RNA recognition motif family protein, expressed	10	IR29
Os.4614.1.S1_a_at	LOC_Os02g40260	Leucine Rich Repeat family protein, expressed	1	IR29
Os.46290.1.A1_at	LOC_Os03g22400	Ulp1 protease family, C-terminal catalytic domain containing protein, expressed	1	IR29
Os.46325.1.A1_at	LOC_Os01g07390	Zinc finger, C3HC4 type family protein, expressed	9	IR29
Os.4639.1.S1_at	LOC_Os08g15030	Aspartate carbamoyltransferase 3, chloroplast precursor, putative, expressed	5	IR29
Os.46466.1.S1_at	LOC_Os10g18890	HNH endonuclease family protein, expressed	9	IR29
Os.46494.1.S1_at	LOC_Os10g26690	ribosomal protein L32 containing protein, expressed	2	IR29
Os.46498.1.S1_at	LOC_Os10g11310	expressed protein	9	IR29
Os.46526.1.S1_at	LOC_Os10g16990	expressed protein	4	IR29
Os.46536.1.S1_at	LOC_Os10g26540	expressed protein	6	IR29
Os.46580.1.S1_at	LOC_Os10g19200	retrotransposon protein, putative, Ty3-gypsy subclass, expressed	10	IR29
Os.4661.1.S1_at	LOC_Os06g47890	ZEITLUPE, putative, expressed	1	IR29
Os.46706.1.S1_x_at	LOC_Os10g15390	expressed protein	10	IR29
Os.46782.1.S1_at	LOC_Os11g07460	TCP family transcription factor containing protein, expressed	4	IR29
Os.4683.2.S1_at	LOC_Os01g42520	expressed protein	3	IR29
Os.46987.1.S1_x_at	LOC_Os07g46480	Eukaryotic aspartyl protease family protein, expressed	3	IR29
Os.47000.1.S1_x_at	LOC_Os12g16350	3-hydroxyisobutyryl-coenzyme A hydrolase, putative, expressed	10	IR29
Os.4732.1.S1_at	LOC_Os08g06430	NADH:ubiquinone oxidoreductase 13 kD-like subunit, putative, expressed	0	IR29
Os.47329.1.A1_at	LOC_Os05g11900	hypothetical protein	10	IR29
Os.47427.1.S1_at	LOC_Os04g36780	expressed protein	10	IR29
Os.47447.1.S1_at	LOC_Os06g06730	early nodule-specific-like protein ENOD8 gene, putative, expressed	10	IR29
Os.47452.1.A1_at	LOC_Os08g27240	expressed protein	6	IR29
Os.47492.1.A1_s_at	LOC_Os02g40430	HEAT repeat family protein, expressed	4	IR29
Os.4765.1.S1_s_at	LOC_Os02g12800	Elongation factor 1-gamma, putative, expressed	4	IR29
Os.47784.1.S1_at	LOC_Os06g45340	FK506-binding protein, putative, expressed	9	IR29

Os.47902.1.S1_at	LOC_Os11g39290	Leucine Rich Repeat family protein, expressed	8	IR29
Os.47929.1.S1_at	LOC_Os09g32200	Mitochondrial carrier protein, expressed	4	IR29
Os.47996.1.A1_at	LOC_Os01g36390	MCM2/3/5 family protein, expressed	8	IR29
Os.48029.1.S1_at	LOC_Os10g33820	expressed protein	4	IR29
Os.48200.1.S1_x_at	LOC_Os09g20590	expressed protein	10	IR29
Os.4824.1.A1_s_at	LOC_Os10g09870	TB2/DP1, HVA22 family protein, expressed	6	IR29
Os.48459.1.A1_at	LOC_Os11g06740	SNELIPTRC, putative, expressed	2	IR29
Os.48621.1.S1_at	LOC_Os02g17190	Myb-like DNA-binding domain containing protein, expressed	1	IR29
Os.4875.1.S1_at	LOC_Os01g69100	expressed protein	3	IR29
Os.48988.1.S1_at	LOC_Os07g47710	60S ribosomal protein L22-2, putative, expressed	8	IR29
Os.49088.1.S1_at	LOC_Os04g44890	tRNA-dihydrouridine synthase 2, putative, expressed	4	IR29
Os.49088.1.S1_at	LOC_Os04g44890	tRNA-dihydrouridine synthase 2, putative, expressed	5	IR29
Os.49109.1.S1_at	LOC_Os08g38850	CRAL/TRIO, N-terminus family protein, expressed	9	IR29
Os.49160.1.S1_at	LOC_Os04g40300	transglutaminase, putative, expressed	10	IR29
Os.49161.1.S1_s_at	LOC_Os05g15510	Cellulase containing protein, expressed	9	IR29
Os.49170.1.S1_at	LOC_Os03g29680	EARLY flowering 4 protein, putative, expressed	6	IR29
Os.49192.1.S1_at	LOC_Os12g17530	expressed protein	0	IR29
Os.49256.1.S1_at	LOC_Os08g14490	4'-phosphopantetheinyl transferase superfamily protein, expressed	6	IR29
Os.49303.1.S1_a_at	LOC_Os04g44710	C-terminal domain phosphatase-like 1, putative, expressed	0	IR29
Os.49344.1.S1_at	LOC_Os01g01570	kinesin heavy chain, putative, expressed	10	IR29
Os.49402.1.S1_at	LOC_Os10g17630	B3 DNA binding domain containing protein, expressed	7	IR29
Os.49459.1.S1_at	LOC_Os11g18170	phospholipid hydroperoxide glutathione peroxidase, putative, expressed	9	IR29
Os.49469.1.S1_at	LOC_Os02g56110	gamma-adaptin 1, putative, expressed	10	IR29
Os.49529.1.S1_at	LOC_Os06g43810	expressed protein	9	IR29
Os.4953.2.S1_x_at	LOC_Os01g58080	membrane-associated salt-inducible protein, putative, expressed	8	IR29
Os.49531.1.S1_at	LOC_Os04g10400	Electron transfer flavoprotein beta-subunit, putative, expressed	8	IR29
Os.49571.1.S1_at	LOC_Os03g15530	expressed protein	9	IR29
Os.49581.1.S1_at	LOC_Os06g40730	expressed protein	8	IR29
Os.49694.1.S1_at	LOC_Os03g62370	WD40-like Beta Propeller Repeat family protein, expressed	7	IR29
Os.49738.1.S1_at	LOC_Os06g01640	Protein arginine N-methyltransferase, putative, expressed	5	IR29
Os.49749.1.S1_at	LOC_Os04g40090	Zinc finger, ZZ type family protein, expressed	2	IR29
Os.49794.1.S1_at	LOC_Os07g06970	HEN1, putative, expressed	10	IR29
Os.49870.1.S1_at	LOC_Os02g06400	Plant protein family protein, expressed	10	IR29
Os.50014.1.S1_at	LOC_Os03g38050	Galactosyltransferase family protein, expressed	2	IR29
Os.50224.1.S1_at	LOC_Os01g69110	expressed protein	8	IR29
Os.5059.1.S1_at	LOC_Os07g44400	kinesin motor protein, putative, expressed	1	IR29
Os.50757.1.S2_at	LOC_Os03g53500	Helicase conserved C-terminal domain containing protein, expressed	2	IR29
Os.50782.1.S1_a_at	LOC_Os12g04740	expressed protein	8	IR29
Os.50909.2.S1_s_at	LOC_Os03g55120	Plastocyanin-like domain containing protein, expressed	6	IR29
Os.50913.1.S1_at	LOC_Os07g42080	expressed protein	10	IR29
Os.50948.1.S1_at	LOC_Os09g12230	Ubiquitin-conjugating enzyme E2-17 kDa, putative, expressed	2	IR29

Os.50956.1.S1_at	LOC_Os03g27040	heavy metal-associated domain containing protein, expressed	10	IR29
Os.51214.1.S1_x_at	LOC_Os09g33520	expressed protein	10	IR29
Os.5129.2.S1_a_at	LOC_Os02g07680	Cytochrome P450 97B2, putative, expressed	3	IR29
Os.51302.1.A1_at	LOC_Os04g08340	signal peptidase I family protein, expressed	8	IR29
Os.5158.1.S1_at	LOC_Os06g06370	expressed protein	9	IR29
Os.51597.1.S1_at	LOC_Os01g68598	expressed protein	2	IR29
Os.51649.1.S1_at	LOC_Os04g33260	expressed protein	10	IR29
Os.51724.1.S1_at	LOC_Os04g40720	expressed protein	3	IR29
Os.5174.1.S1_at	LOC_Os01g67530	AMP-binding enzyme family protein, expressed	0	IR29
Os.5186.1.S1_at	LOC_Os04g33390	prephenate dehydratase family protein, expressed	1	IR29
Os.52025.1.S1_at	LOC_Os11g19730	expressed protein	5	IR29
Os.52093.1.S1_at	LOC_Os09g31446	expressed protein	9	IR29
Os.52099.1.S1_at	LOC_Os06g03059	expressed protein	10	IR29
Os.52156.1.S2_at	LOC_Os11g20360	Leucine Rich Repeat family protein, expressed	0	IR29
Os.52178.1.S1_at	LOC_Os08g07940	NB-ARC domain containing protein	8	IR29
Os.52190.1.S1_at	LOC_Os11g27660	hypothetical protein	9	IR29
Os.52202.1.S1_at	LOC_Os03g22450	Rad9 family protein, expressed	9	IR29
Os.52245.1.S1_at	LOC_Os06g02830	expressed protein	4	IR29
Os.5243.1.S1_at	LOC_Os02g55320	Two-component response regulator ARR12, putative, expressed	9	IR29
Os.52446.1.S1_at	LOC_Os04g11450	F-box domain containing protein, expressed	3	IR29
Os.52482.1.S1_at	LOC_Os07g36630	Cellulose synthase family protein, expressed	2	IR29
Os.52483.1.S1_at	LOC_Os06g19380	transporter, putative, expressed	9	IR29
Os.52634.2.S1_x_at	LOC_Os09g15639	expressed protein	9	IR29
Os.52636.1.S1_at	LOC_Os06g02590	START domain-containing protein, putative, expressed	6	IR29
Os.5268.1.S1_at	LOC_Os12g01580	MATE efflux family protein, expressed	8	IR29
Os.5273.2.S1_x_at	LOC_Os06g02600	DAG protein, chloroplast precursor, putative, expressed	7	IR29
Os.5283.1.S1_at	LOC_Os11g01510	Ubiquitin-activating enzyme E1 2, putative, expressed	6	IR29
Os.52844.1.S1_at	LOC_Os03g53510	transducin family protein, putative, expressed	8	IR29
Os.52921.1.S1_at	LOC_Os08g14760	4-coumarate-CoA ligase 1, putative, expressed	7	IR29
Os.52945.1.S1_at	LOC_Os12g02950	pentatricopeptide, putative, expressed	1	IR29
Os.53062.1.S1_at	LOC_Os05g03550	Myb-like DNA-binding domain containing protein, expressed	10	IR29
Os.53189.1.S1_at	LOC_Os08g07430	expressed protein	8	IR29
Os.53309.1.S1_at	LOC_Os07g47650	Kelch motif family protein, expressed	8	IR29
Os.53388.2.S1_at	LOC_Os08g40820	expressed protein	1	IR29
Os.5344.1.S1_at	LOC_Os06g44140	Endomembrane protein 70 containing protein, expressed	9	IR29
Os.53565.1.A1_s_at	LOC_Os03g22400	Ulp1 protease family, C-terminal catalytic domain containing protein, expressed	6	IR29
Os.53575.1.S1_at	LOC_Os03g21970	bHLH transcription factor bHLH033, putative, expressed	8	IR29
Os.53609.1.S1_s_at	LOC_Os01g10490	expressed protein	0	IR29
Os.53612.1.S1_at	LOC_Os06g14450	Exo70 exocyst complex subunit family protein, expressed	6	IR29
Os.53615.1.S1_at	LOC_Os07g01130	expressed protein	9	IR29
Os.5376.1.S1_at	LOC_Os07g01910	expressed protein	9	IR29

Os.53795.1.S1_at	LOC_Os03g08840	expressed protein	8	IR29
Os.53799.1.S1_at	LOC_Os06g12060	mTERF family protein, expressed	10	IR29
Os.53930.1.S1_at	LOC_Os09g12730	RNA recognition motif family protein, expressed	7	IR29
Os.5395.1.S1_at	LOC_Os05g12260	CAPIP1, putative, expressed	2	IR29
Os.53968.1.S1_at	LOC_Os02g56310	Protein kinase domain containing protein, expressed	8	IR29
Os.5423.1.S1_at	LOC_Os03g16690	Oxysterol-binding protein, expressed	6	IR29
Os.54247.1.S1_at	LOC_Os01g67550	FAD binding domain containing protein	6	IR29
Os.54276.1.A1_at	LOC_Os09g32130	expressed protein	10	IR29
Os.54299.1.S1_at	LOC_Os06g10880	bZIP transcription factor family protein, expressed	8	IR29
Os.54303.1.S1_at	LOC_Os04g43300	BRCA1 C Terminus domain containing protein, expressed	4	IR29
Os.54312.1.S1_at	LOC_Os11g05570	ribonuclease HI large subunit, putative, expressed	3	IR29
Os.54509.1.S1_at	LOC_Os09g19650	fatty acid elongase, putative, expressed	10	IR29
Os.54609.1.S1_at	LOC_Os03g01090	expressed protein	7	IR29
Os.54857.1.S1_at	LOC_Os02g56750	F-box domain containing protein, expressed	5	IR29
Os.54858.1.S1_at	LOC_Os10g40660	Zinc finger, C2H2 type family protein, expressed	7	IR29
Os.5511.1.S1_s_at	LOC_Os06g12250	Protein SUR2, putative, expressed	7	IR29
Os.55230.1.S1_at	LOC_Os09g33882	expressed protein	8	IR29
Os.55282.1.S1_at	LOC_Os09g30140	expressed protein	10	IR29
Os.55477.1.S1_at	LOC_Os06g28740	expressed protein	6	IR29
Os.55633.1.A1_at	LOC_Os08g14360	expressed protein	7	IR29
Os.5571.1.S1_at	LOC_Os02g01240	Ankyrin-1, putative, expressed	7	IR29
Os.55744.1.S1_at	LOC_Os04g43130	Transcriptional corepressor LEUNIG, putative, expressed	3	IR29
Os.55784.1.S1_at	LOC_Os02g39990	meprin and TRAF homology domain-containing protein, putative, expressed	8	IR29
Os.55958.1.S1_at	LOC_Os12g01480	phosphatidate cytidylyltransferase family protein, expressed	7	IR29
Os.56188.1.S1_at	LOC_Os02g38490	expressed protein	1	IR29
Os.5620.1.S1_at	LOC_Os05g01320	expressed protein	8	IR29
Os.5620.2.S1_s_at	LOC_Os01g02180	expressed protein	5	IR29
Os.56224.1.S1_at	LOC_Os01g02010	expressed protein	9	IR29
Os.56233.1.S1_s_at	LOC_Os09g30010	Fasciclin-like arabinogalactan protein 1 precursor, putative, expressed	6	IR29
Os.56238.1.S1_at	LOC_Os11g16560	expressed protein	0	IR29
Os.56322.1.S1_at	LOC_Os02g13310	Associated with HOX family protein, expressed	9	IR29
Os.56337.1.S1_at	LOC_Os11g19210	Glycosyl hydrolase family 3 C terminal domain containing protein, expressed	4	IR29
Os.5666.1.S1_at	LOC_Os02g15580	Cyclic nucleotide-gated ion channel 1, putative, expressed	2	IR29
Os.5713.1.S1_at	LOC_Os11g31650	hypothetical protein	0	IR29
Os.57154.1.S1_at	LOC_Os02g09780	expressed protein	2	IR29
Os.5720.1.S1_s_at	LOC_Os04g58620	K+ efflux antiporter, putative, expressed	1	IR29
Os.57207.1.S1_s_at	LOC_Os12g40760	expressed protein	6	IR29
Os.57313.1.S1_at	LOC_Os12g31710	universal stress protein family protein, expressed	8	IR29
Os.57477.1.S1_x_at	LOC_Os11g47760	Heat shock cognate 70 kDa protein, putative, expressed	8	IR29
Os.57557.1.S1_at	LOC_Os09g20980	Zinc finger, C3HC4 type family protein, expressed	1	IR29
Os.57573.1.S1_at	LOC_Os11g06130	PHD-finger family protein, expressed	10	IR29

Os.5788.1.S1_at	LOC_Os02g48180	PDZ domain family protein, expressed	10	IR29
Os.5902.1.S2_at	LOC_Os04g47180	expressed protein	10	IR29
Os.5908.1.S1_at	LOC_Os01g47780	Fasciclin domain containing protein, expressed	6	IR29
Os.5927.1.S1_at	LOC_Os03g63540	expressed protein	5	IR29
Os.5947.1.S1_at	LOC_Os01g73470	Growth inhibition and differentiation-related protein 88 homolog, putative, expressed	3	IR29
Os.5964.1.S1_at	LOC_Os06g12110	mTERF family protein, expressed	8	IR29
Os.5974.1.A1_at	LOC_Os09g29430	anion transporter family protein, expressed	0	IR29
Os.5998.1.S1_at	LOC_Os12g02120	Dual specificity phosphatase, catalytic domain containing protein, expressed	7	IR29
Os.6011.1.S1_at	LOC_Os10g16974	Flavonoid 3'-monooxygenase, putative, expressed	9	IR29
Os.6014.2.S1_at	LOC_Os01g04300	endo-1,4-beta-xylanase, putative, expressed	5	IR29
Os.6028.1.S1_at	LOC_Os04g40940	Mitotic spindle checkpoint protein MAD2, putative, expressed	10	IR29
Os.6118.1.S1_at	LOC_Os11g05700	ABC transporter family protein, expressed	6	IR29
Os.6169.1.S1_at	LOC_Os01g72320	zinc finger protein, putative, expressed	1	IR29
Os.6193.1.S1_at	LOC_Os04g40060	transposon protein, putative, unclassified, expressed	6	IR29
Os.6208.1.S1_at	LOC_Os07g01140	BTB/POZ domain containing protein, expressed	5	IR29
Os.6226.1.S1_at	LOC_Os11g11370	expressed protein	6	IR29
Os.6234.1.S1_s_at	LOC_Os04g41040	DNA-directed RNA polymerases II 24 kDa polypeptide, putative, expressed	5	IR29
Os.6282.1.S1_at	LOC_Os02g57540	Ribosomal L28e protein family protein, expressed	4	IR29
Os.6411.1.S1_a_at	LOC_Os07g42260	Proteasome subunit alpha type 6, putative, expressed	8	IR29
Os.6438.1.S1_a_at	LOC_Os01g31220	expressed protein	3	IR29
Os.6438.2.S1_x_at	LOC_Os01g31220	expressed protein	8	IR29
Os.6450.1.S1_at	LOC_Os07g09190	1-deoxy-D-xylulose-5-phosphate synthase, chloroplast precursor, putative, expressed	9	IR29
Os.6557.1.S1_s_at	LOC_Os08g07300	haloacid dehalogenase-like hydrolase family protein, putative, expressed	0	IR29
Os.6597.1.S1_at	LOC_Os01g01960	Paired amphipathic helix repeat family protein, expressed	2	IR29
Os.6638.1.S1_x_at	LOC_Os01g57770	hydrolase, alpha/beta fold family protein, expressed	10	IR29
Os.6641.1.S1_at	LOC_Os11g16320	expressed protein	6	IR29
Os.6664.1.S1_at	LOC_Os05g47700	nonspecific lipid-transfer protein AKCS9 precursor, putative, expressed	10	IR29
Os.6726.1.S1_x_at	LOC_Os04g38630	Helicase conserved C-terminal domain containing protein, expressed	0	IR29
Os.6786.1.S1_a_at	LOC_Os10g40720	Beta-expansin 1a precursor, putative, expressed	5	IR29
Os.6860.1.S1_at	LOC_Os11g21990	expressed protein	5	IR29
Os.6980.1.S1_at	LOC_Os12g38210	Spotted leaf protein 11, putative, expressed	1	IR29
Os.7012.1.S1_at	LOC_Os01g72000	Armadillo/beta-catenin-like repeat family protein, expressed	9	IR29
Os.7034.1.S1_at	LOC_Os03g22420	AAA-type ATPase family protein, putative, expressed	5	IR29
Os.7064.1.S1_s_at	LOC_Os02g06360	KpsF/GutQ family protein, expressed	8	IR29
Os.7085.2.S1_x_at	LOC_Os07g47700	NAD-dependent epimerase/dehydratase family protein, putative, expressed	6	IR29
Os.7089.2.S1_at	LOC_Os10g16640	expressed protein	5	IR29
Os.7133.1.S1_at	LOC_Os01g34700	Dienelactone hydrolase family protein, expressed	1	IR29
Os.7218.2.S1_at	LOC_Os02g01150	D-isomer specific 2-hydroxyacid dehydrogenase, NAD binding domain containing protein, expressed	9	IR29
Os.7277.1.S1_s_at	LOC_Os03g44670	expressed protein	8	IR29
Os.7306.1.S1_at	LOC_Os03g01200	SNF2P, putative, expressed	9	IR29
Os.7307.2.S1_x_at	LOC_Os01g33800	DnaJ domain containing protein, expressed	6	IR29

Os.7309.2.S1_at	LOC_Os12g01360	expressed protein	7	IR29
Os.7317.2.S1_at	LOC_Os01g68589	Protease inhibitor/seed storage/LTP family protein, expressed	6	IR29
Os.7323.1.S1_at	LOC_Os01g40340	SIT4 phosphatase-associated family protein, putative, expressed	4	IR29
Os.7419.1.S1_a_at	LOC_Os02g58490	PINHEAD protein, putative, expressed	9	IR29
Os.7465.1.S1_at	LOC_Os05g28980	expressed protein	3	IR29
Os.7513.1.S1_at	LOC_Os11g39220	Acyl-coenzyme A oxidase 2, peroxisomal precursor, putative, expressed	2	IR29
Os.7522.1.S1_at	LOC_Os02g40890	expressed protein	4	IR29
Os.7532.1.S1_at	LOC_Os02g20380	expressed protein	2	IR29
Os.7558.1.S1_s_at	LOC_Os03g49150	Yippee, putative, expressed	2	IR29
Os.7574.1.S1_s_at	LOC_Os02g05510	GATA transcription factor 25, putative, expressed	6	IR29
Os.7576.1.S1_at	LOC_Os04g32980	expressed protein	9	IR29
Os.7584.1.S1_at	LOC_Os04g02000	Zn-finger in Ran binding protein and others containing protein, expressed	4	IR29
Os.7599.1.S1_at	LOC_Os06g40710	transfactor, putative, expressed	10	IR29
Os.7668.2.S1_x_at	LOC_Os04g17480	transposon protein, putative, unclassified	5	IR29
Os.7680.1.S1_at	LOC_Os07g04020	expressed protein	10	IR29
Os.7733.1.S2_at	LOC_Os01g68524	pentatricopeptide, putative, expressed	7	IR29
Os.7792.1.S1_at	LOC_Os04g37690	RNA recognition motif family protein, expressed	9	IR29
Os.7809.1.S1_at	LOC_Os09g19890	expressed protein	1	IR29
Os.7811.1.S1_at	LOC_Os06g12220	HVA22-like protein a, putative, expressed	6	IR29
Os.7865.1.S1_at	LOC_Os06g15400	expressed protein	10	IR29
Os.7900.1.S1_s_at	LOC_Os03g26970	Proteasome subunit alpha type 2, putative, expressed	10	IR29
Os.7901.1.S1_at	LOC_Os04g58850	Harpin-induced protein 1 containing protein, expressed	9	IR29
Os.7905.1.S1_at	LOC_Os01g11570	GDSL-like Lipase/Acylhydrolase family protein, expressed	9	IR29
Os.7907.2.S1_x_at	LOC_Os07g46460	Ferredoxin-dependent glutamate synthase, chloroplast precursor, putative, expressed	10	IR29
Os.7927.1.S1_a_at	LOC_Os10g17454	RNA recognition motif family protein, expressed	0	IR29
Os.7927.1.S1_a_at	LOC_Os10g17454	RNA recognition motif family protein, expressed	1	IR29
Os.7928.1.S1_at	LOC_Os03g48970	CCAAT-binding transcription factor subunit B family protein, expressed	9	IR29
Os.7948.1.S1_a_at	LOC_Os01g17150	expressed protein	6	IR29
Os.7952.1.S1_at	LOC_Os03g25980	Polypyrimidine tract-binding protein homolog 1, putative, expressed	9	IR29
Os.8019.1.S1_at	LOC_Os01g49830	DNA-binding protein RAV1, putative, expressed	7	IR29
Os.8021.1.S1_x_at	LOC_Os04g47250	Cytochrome P450 86A2, putative, expressed	5	IR29
Os.8042.1.S1_at	LOC_Os02g13110	Serine/threonine protein phosphatase 2A, 55 kDa regulatory subunit B,beta isoform, putative, expresse	9	IR29
Os.8132.1.S1_at	LOC_Os02g57370	Zinc finger DHHC domain containing protein 15 homolog, putative, expressed	10	IR29
Os.8139.1.S1_at	LOC_Os09g29490	Peroxidase 17 precursor, putative, expressed	5	IR29
Os.8200.2.S1_x_at	LOC_Os03g63770	Poly polymerase catalytic domain containing protein, expressed	6	IR29
Os.8225.1.S1_at	LOC_Os03g15940	Pollen-specific protein SF3, putative, expressed	5	IR29
Os.8239.1.S1_at	LOC_Os06g13680	senescence-associated protein, putative, expressed	5	IR29
Os.8372.1.S1_at	LOC_Os03g01750	protein tyrosine phosphatase, putative, expressed	10	IR29
Os.8442.1.S1_at	LOC_Os04g39900	Non-cyanogenic beta-glucosidase precursor, putative, expressed	2	IR29
Os.8453.1.S1_a_at	LOC_Os06g09890	Smr domain containing protein, expressed	3	IR29
Os.8464.1.A1_at	LOC_Os05g12140	Leucine Rich Repeat family protein, expressed	9	IR29

Os.8469.1.S1_at	LOC_Os09g34140	expressed protein	4	IR29
Os.8493.1.A1_at	LOC_Os06g43430	Cytochrome P450 family protein, expressed	4	IR29
Os.8527.1.S1_at	LOC_Os10g20240	expressed protein	9	IR29
Os.8565.1.S1_at	LOC_Os02g32490	Acetyl-coenzyme A synthetase, putative, expressed	4	IR29
Os.8602.2.S1_at	LOC_Os02g21270	hypothetical protein	8	IR29
Os.8611.1.S1_at	LOC_Os05g29760	Ferrochelatase II, chloroplast precursor, putative, expressed	9	IR29
Os.8619.1.S1_at	LOC_Os04g36859	signal peptidase complex 25 kDa subunit, putative, expressed	9	IR29
Os.8653.1.S1_at	LOC_Os02g32610	Protein kinase domain containing protein, expressed	9	IR29
Os.8727.1.S1_at	LOC_Os03g63140	Nitrate-induced NOI protein, expressed	3	IR29
Os.8734.1.S1_a_at	LOC_Os09g35810	NC domain-containing protein, putative, expressed	10	IR29
Os.8739.3.S1_x_at	LOC_Os01g60440	HEAT repeat-containing protein, putative, expressed	3	IR29
Os.874.1.S1_at	LOC_Os01g14610	DNA replication complex GINS protein PSF2, putative, expressed	1	IR29
Os.8743.1.S1_at	LOC_Os07g46430	expressed protein	2	IR29
Os.8760.1.S1_a_at	LOC_Os09g36600	Nodulin-like family protein, expressed	5	IR29
Os.8814.1.S1_at	LOC_Os04g11880	Vacuolar assembly protein VPS41 homolog, putative, expressed	4	IR29
Os.8827.1.S1_at	LOC_Os01g60740	Nonspecific lipid-transfer protein precursor, putative, expressed	8	IR29
Os.8874.1.S1_at	LOC_Os11g08080	SWIRM domain containing protein, expressed	7	IR29
Os.8940.1.S1_at	LOC_Os06g12030	Domain found in Dishevelled, Egl-10, and Pleckstrin family protein, expressed	3	IR29
Os.9003.1.S1_at	LOC_Os11g43620	small nuclear ribonucleoprotein F, putative, expressed	5	IR29
Os.9021.1.S1_at	LOC_Os03g22430	ATP-dependent Clp protease proteolytic subunit, putative, expressed	5	IR29
Os.9029.1.S1_at	LOC_Os01g15350	Zinc finger CCCH type domain containing protein ZFN-like 3, putative, expressed	10	IR29
Os.9048.1.S1_at	LOC_Os03g63590	Cleavage and polyadenylation specificity factor, 73 kDa subunit, putative, expressed	5	IR29
Os.9072.1.S1_at	LOC_Os11g30360	expressed protein	8	IR29
Os.9099.1.S1_at	LOC_Os07g27150	Trafficking protein particle complex subunit 4, putative, expressed	9	IR29
Os.9122.1.S1_at	LOC_Os02g39630	iron-stress related protein, putative, expressed	9	IR29
Os.9172.2.S1_x_at	LOC_Os02g02400	Catalase isozyme A, putative, expressed	10	IR29
Os.9195.1.S1_at	LOC_Os01g43370	expressed protein	5	IR29
Os.9234.1.S1_at	LOC_Os07g46540	HEAT repeat family protein, expressed	2	IR29
Os.9247.2.S1_x_at	LOC_Os06g07230	Protein kinase APK1B, chloroplast precursor, putative, expressed	3	IR29
Os.9293.1.S1_at	LOC_Os04g40040	Copper amine oxidase, enzyme domain containing protein, expressed	10	IR29
Os.9382.1.S1_at	LOC_Os06g02780	Eukaryotic aspartyl protease family protein, expressed	8	IR29
Os.9390.1.S1_at	LOC_Os02g32750	Glycosyltransferase 5, putative, expressed	1	IR29
Os.94.1.S1_at	LOC_Os01g15979	gb protein, putative, expressed	9	IR29
Os.9449.1.S1_at	LOC_Os08g33920	60S ribosomal protein L34, putative, expressed	4	IR29
Os.9489.1.S1_x_at	LOC_Os08g26870	wound-responsive protein, putative, expressed	10	IR29
Os.9510.1.S1_s_at	LOC_Os06g12690	DCN1-like protein 2, putative, expressed	8	IR29
Os.9512.1.A1_at	LOC_Os04g43130	Transcriptional corepressor LEUNIG, putative, expressed	4	IR29
Os.9720.1.S1_at	LOC_Os08g14770	aminotransferase class III family protein, putative, expressed	6	IR29
Os.9721.1.S1_at	LOC_Os08g01310	MAP3K-like protein kinase, putative, expressed	9	IR29
Os.9761.1.S1_at	LOC_Os06g40740	expressed protein	9	IR29
Os.9773.1.S1_at	LOC_Os01g59060	ribosomal protein L34 containing protein, expressed	9	IR29

Os.9792.1.S1_at	LOC_Os02g05890	expressed protein	9	IR29
Os.9899.1.S1_at	LOC_Os06g10690	C1-like domain containing protein, expressed	4	IR29
Os.9960.1.S1_at	LOC_Os06g07540	expressed protein	9	IR29
Os.9992.1.S1_a_at	LOC_Os01g58380	3-hydroxybutyryl-CoA dehydrogenase, putative, expressed	5	IR29
Os.9996.2.S1_at	LOC_Os11g10980	pyruvate kinase family protein, expressed	3	IR29
Os.9998.1.S1_at	LOC_Os01g68324	Ribophorin II family protein, expressed	2	IR29
OsAffx.12042.1.S1_s_at	LOC_Os02g14860	KE2 family protein, expressed	1	IR29
OsAffx.12889.1.S1_s_at	LOC_Os03g17060	RNA-binding protein, putative, expressed	10	IR29
OsAffx.12991.1.S1_s_at	LOC_Os03g22320	Utp14 protein, expressed	6	IR29
OsAffx.13351.1.S1_at	LOC_Os03g44800	expressed protein	4	IR29
OsAffx.13484.1.S1_at	LOC_Os03g53970	expressed protein	8	IR29
OsAffx.13583.1.S1_at	LOC_Os03g62420	expressed protein	4	IR29
OsAffx.14155.1.S1_at	LOC_Os04g35420	ATP-dependent DNA helicase, RecQ family protein, expressed	1	IR29
OsAffx.15432.1.S1_at	LOC_Os06g14750	Phosphatidylinositol-4-phosphate 5-Kinase family protein, expressed	0	IR29
OsAffx.17471.1.S1_at	LOC_Os08g42210	cation exchanger, putative	3	IR29
OsAffx.17990.1.S1_at	LOC_Os09g33920	expressed protein	10	IR29
OsAffx.17991.1.S1_at	LOC_Os09g34090	NAD dependent epimerase/dehydratase family protein, expressed	1	IR29
OsAffx.18282.1.S1_s_at	LOC_Os10g18070	expressed protein	6	IR29
OsAffx.18950.1.S1_x_at	LOC_Os11g19810	PWWP domain containing protein, expressed	4	IR29
OsAffx.19804.1.S1_at	LOC_Os12g24580	negatively light-regulated protein, putative, expressed	0	IR29
OsAffx.23355.1.S1_s_at	LOC_Os01g23630	Transcription initiation factor IID, 18kD subunit family protein	4	IR29
OsAffx.23435.1.S1_at	LOC_Os01g28730	serine/threonine protein kinase, putative, expressed	1	IR29
OsAffx.23960.1.S1_s_at	LOC_Os01g67600	esterase, putative, expressed	2	IR29
OsAffx.24132.1.S1_at	LOC_Os02g06584	Zinc finger C-x8-C-x5-C-x3-H type family protein, expressed	0	IR29
OsAffx.24560.1.S1_s_at	LOC_Os02g33730	Ubiquinol-cytochrome C reductase hinge protein, expressed	5	IR29
OsAffx.24579.1.S1_x_at	LOC_Os02g34850	CW-type Zinc Finger family protein, expressed	5	IR29
OsAffx.24656.1.S1_s_at	LOC_Os02g40000	Sedlin, N-terminal conserved region family protein, expressed	9	IR29
OsAffx.24660.1.S1_x_at	LOC_Os02g40410	expressed protein	3	IR29
OsAffx.25285.1.S1_at	LOC_Os03g29770	EF hand family protein, expressed	7	IR29
OsAffx.25290.1.S1_at	LOC_Os03g29960	expressed protein	6	IR29
OsAffx.26298.1.S1_at	LOC_Os04g32970	OTU-like cysteine protease family protein, putative, expressed	2	IR29
OsAffx.26399.1.S1_at	LOC_Os04g39970	vacuolar sorting protein 9 domain-containing protein, putative, expressed	2	IR29
OsAffx.26803.1.S1_at	LOC_Os05g09520	IQ calmodulin-binding motif family protein, expressed	9	IR29
OsAffx.27689.1.S1_s_at	LOC_Os06g17870	nitrate-induced NOI protein, putative	3	IR29
OsAffx.27908.1.S1_at	LOC_Os01g12860	Myb-like DNA-binding domain containing protein, expressed	3	IR29
OsAffx.28026.1.S1_at	LOC_Os06g40590	expressed protein	6	IR29
OsAffx.29124.1.S1_at	LOC_Os08g09920	F-box domain containing protein, expressed	8	IR29
OsAffx.29166.1.S1_at	LOC_Os08g12850	pentatricopeptide, putative, expressed	7	IR29
OsAffx.29394.1.S1_s_at	LOC_Os08g28190	Actin-like protein 2, putative, expressed	7	IR29
OsAffx.31542.1.S1_x_at	LOC_Os12g01740	Protein kinase domain containing protein, expressed	6	IR29
OsAffx.31642.1.S1_s_at	LOC_Os12g08230	expressed protein	1	IR29

OsAffx.3363.1.S1_at	LOC_Os03g29460	60S ribosomal protein L27a-3, putative, expressed	6	IR29
OsAffx.3763.1.S1_s_at	LOC_Os04g10420	expressed protein	7	IR29
OsAffx.4094.1.S1_at	LOC_Os04g45000	oxidoreductase, short chain dehydrogenase/reductase family protein, expressed	0	IR29
OsAffx.5113.1.S1_at	LOC_Os06g45310	SBP domain containing protein, expressed	10	IR29
OsAffx.5147.1.S1_at	LOC_Os06g48720	Cadmium/zinc-transporting ATPase 4, putative, expressed	5	IR29
OsAffx.5503.1.S1_at	LOC_Os07g32790	expressed protein	10	IR29
OsAffx.5607.1.S1_x_at	LOC_Os07g42280	von Willebrand factor type A domain containing protein, expressed	2	IR29
OsAffx.5613.1.S1_x_at	LOC_Os07g42632	dihydropteroate synthase family protein, expressed	3	IR29
OsAffx.5702.1.S1_s_at	LOC_Os08g04560	Pyridoxal-dependent decarboxylase conserved domain containing protein, expressed	9	IR29
OsAffx.5926.1.S1_at	LOC_Os08g25820	transfactor, putative, expressed	7	IR29
OsAffx.5976.1.S1_at	LOC_Os08g29400	F-box domain containing protein, expressed	4	IR29
OsAffx.6701.1.S1_s_at	LOC_Os10g19190	Got1-like family protein, expressed	3	IR29
OsAffx.7123.1.S1_at	LOC_Os11g16430	diphthamide biosynthesis protein 2 containing protein, expressed	7	IR29
OsAffx.7471.1.S1_x_at	LOC_Os12g03500	expressed protein	3	IR29
Os.10028.1.S1_x_at	LOC_Os04g29920	Reticulon family protein, expressed	8	Pokkali
Os.1005.1.S1_at	LOC_Os01g01710	1-deoxy-D-xylulose 5-phosphate reductoisomerase, chloroplast precursor, putative, expressed	9	Pokkali
Os.10107.2.S1_x_at	LOC_Os04g58700	leucine-rich repeat transmembrane protein kinase, putative, expressed	8	Pokkali
Os.10132.1.S1_at	LOC_Os09g36300	Lon protease homolog 1, mitochondrial precursor, putative, expressed	10	Pokkali
Os.10153.1.S1_at	LOC_Os12g36180	expressed protein	0	Pokkali
Os.10155.1.S1_at	LOC_Os05g02060	amino acid selective channel protein, putative, expressed	6	Pokkali
Os.10328.1.S1_at	LOC_Os03g55610	Dof domain, zinc finger family protein, expressed	6	Pokkali
Os.10351.1.S1_a_at	LOC_Os08g40530	Calcium-transporting ATPase 9, plasma membrane-type, putative, expressed	2	Pokkali
Os.10473.1.S1_at	LOC_Os12g25690	UDP-glucose 6-dehydrogenase, putative, expressed	5	Pokkali
Os.10491.1.S1_at	LOC_Os03g04690	expressed protein	8	Pokkali
Os.10530.1.S1_at	LOC_Os08g43380	RabGAP/TBC domain-containing protein, putative, expressed	5	Pokkali
Os.10551.1.S1_at	LOC_Os10g32550	Chaperonin CPN60-1, mitochondrial precursor, putative, expressed	10	Pokkali
Os.10566.1.S1_at	LOC_Os02g42320	Proteasome subunit alpha type 2, putative, expressed	6	Pokkali
Os.10612.1.S1_at	LOC_Os03g61960	Ferredoxin-3, chloroplast precursor, putative, expressed	10	Pokkali
Os.10620.1.S1_at	LOC_Os05g07940	lactoylglutathione lyase family protein, putative, expressed	0	Pokkali
Os.10755.1.S1_at	LOC_Os09g19640	holocarboxylase synthetase, putative, expressed	3	Pokkali
Os.10845.1.S1_s_at	LOC_Os03g60370	histidine acid phosphatase family protein, putative, expressed	5	Pokkali
Os.10922.1.S1_at	LOC_Os12g28270	Amidohydrolase family protein, expressed	10	Pokkali
Os.10964.1.S1_at	LOC_Os05g27920	phox domain-containing protein, putative, expressed	7	Pokkali
Os.10970.1.S1_at	LOC_Os03g54920	expressed protein	10	Pokkali
Os.10971.1.S1_at	LOC_Os04g54330	N-terminal acetyltransferase complex ARD1 subunit homolog, putative, expressed	9	Pokkali
Os.11021.2.S1_x_at	LOC_Os05g07700	60S ribosomal protein L10-3, putative, expressed	0	Pokkali
Os.11106.1.S1_at	LOC_Os06g46900	phosphosulfolactate synthase-related protein, putative, expressed	8	Pokkali
Os.11132.1.S1_s_at	LOC_Os08g08120	B-box zinc finger family protein, expressed	8	Pokkali
Os.11185.1.S1_a_at	LOC_Os02g17500	Sugar transporter family protein, expressed	6	Pokkali
Os.11210.1.S1_at	LOC_Os12g37610	ribosomal protein S6 containing protein, expressed	8	Pokkali
Os.11392.2.S1_at	LOC_Os02g07820	expressed protein	10	Pokkali

Os.11513.1.S1_at	LOC_Os12g38140	expressed protein	6	Pokkali
Os.11530.1.S1_at	LOC_Os04g31820	hypothetical protein	7	Pokkali
Os.11568.2.S1_at	LOC_Os12g06610	Uncharacterised protein family containing protein, expressed	0	Pokkali
Os.11590.1.S2_at	LOC_Os03g44010	expressed protein	8	Pokkali
Os.11610.2.S1_at	LOC_Os02g42020	expressed protein	2	Pokkali
Os.11614.1.S1_s_at	LOC_Os03g55730	expressed protein	8	Pokkali
Os.11704.1.S1_at	LOC_Os03g58090	expressed protein	1	Pokkali
Os.11731.1.S1_at	LOC_Os05g28290	Ran-binding protein 1 homolog c, putative, expressed	7	Pokkali
Os.11748.2.S1_a_at	LOC_Os03g62640	RNA-binding protein Luc7-like 2, putative, expressed	4	Pokkali
Os.11750.1.A1_at	LOC_Os02g03230	Nodule membrane protein, putative, expressed	8	Pokkali
Os.11790.1.S1_at	LOC_Os08g42560	Glycyl-tRNA synthetase, putative, expressed	3	Pokkali
Os.11795.1.S1_a_at	LOC_Os03g63950	Plastid-specific 30S ribosomal protein 1, chloroplast precursor, putative, expressed	1	Pokkali
Os.11808.1.S1_at	LOC_Os03g06580	expressed protein	8	Pokkali
Os.11845.1.S1_a_at	LOC_Os12g42550	Methyl-CpG binding domain containing protein, expressed	6	Pokkali
Os.11924.1.S1_at	LOC_Os12g41820	DNAJ heat shock N-terminal domain-containing protein, putative, expressed	2	Pokkali
Os.11988.1.S1_at	LOC_Os08g08820	START domain containing protein, expressed	3	Pokkali
Os.12004.1.S1_at	LOC_Os05g02990	expressed protein	8	Pokkali
Os.12177.1.S1_at	LOC_Os12g31660	transposon protein, putative, CACTA, En/Spm sub-class, expressed	2	Pokkali
Os.12238.2.S1_x_at	LOC_Os02g01560	40S ribosomal protein S4, putative, expressed	8	Pokkali
Os.12264.2.S2_a_at	LOC_Os03g12670	expressed protein	2	Pokkali
Os.12312.1.S1_at	LOC_Os01g69920	histidine kinase 2, putative, expressed	2	Pokkali
Os.12338.1.S1_at	LOC_Os05g42330	secretory carrier membrane protein family protein, putative, expressed	3	Pokkali
Os.12374.1.S1_at	LOC_Os12g42884	5-methyltetrahydropteroyltriglutamate-homocysteine	2	Pokkali
Os.12387.1.S1_at	LOC_Os04g39150	Pathogenesis-related protein Bet v I family protein, expressed	6	Pokkali
Os.12414.1.S1_at	LOC_Os09g36800	3-dehydroquinate synthase, putative, expressed	5	Pokkali
Os.12590.1.S1_at	LOC_Os06g02510	60S ribosomal protein L13-2, putative, expressed	4	Pokkali
Os.12656.1.S1_s_at	LOC_Os06g35200	Leucine Rich Repeat family protein, expressed	9	Pokkali
Os.12705.1.S1_at	LOC_Os03g15890	RNA recognition motif family protein, expressed	0	Pokkali
Os.12705.2.S1_x_at	LOC_Os03g15890	RNA recognition motif family protein, expressed	6	Pokkali
Os.12706.1.S1_at	LOC_Os05g02530	dehydroascorbate reductase, putative, expressed	5	Pokkali
Os.12766.1.S1_at	LOC_Os03g14570	expressed protein	2	Pokkali
Os.12964.1.A1_s_at	LOC_Os03g22800	Tubby protein, putative, expressed	6	Pokkali
Os.12994.1.S1_at	LOC_Os12g38400	Myb-like DNA-binding domain containing protein, expressed	7	Pokkali
Os.1302.1.S1_at	LOC_Os01g10610	BES1/BZR1 homolog protein 4, putative, expressed	0	Pokkali
Os.13468.1.S1_at	LOC_Os03g58820	aminoacyl-tRNA synthetase family, putative, expressed	6	Pokkali
Os.13480.1.S1_at	LOC_Os04g10680	Zinc finger, C3HC4 type family protein, expressed	9	Pokkali
Os.13544.1.S1_x_at	LOC_Os01g37832	GM14633p, putative, expressed	9	Pokkali
Os.13734.1.S1_s_at	LOC_Os08g41460	expressed protein	0	Pokkali
Os.13960.2.S1_x_at	LOC_Os01g73560	Leucine Rich Repeat family protein, expressed	7	Pokkali
Os.14078.1.S1_s_at	LOC_Os10g07210	Hsp20/alpha crystallin family protein, expressed	3	Pokkali
Os.14200.2.S1_x_at	LOC_Os10g24070	expressed protein	2	Pokkali

Os.14296.1.S1_at	LOC_Os09g38759	Dihydroneopterin aldolase, putative, expressed	9	Pokkali
Os.14326.1.S1_at	LOC_Os04g35920	Smg-4/UPF3 family protein, expressed	4	Pokkali
Os.14351.1.A1_at	LOC_Os01g57066	expressed protein	10	Pokkali
Os.14356.1.S1_at	LOC_Os09g35690	Zinc finger, C3HC4 type family protein, expressed	5	Pokkali
Os.14424.1.S1_at	LOC_Os12g29560	DHHC zinc finger domain containing protein, expressed	7	Pokkali
Os.14476.1.S1_a_at	LOC_Os02g07230	Porphobilinogen deaminase, chloroplast precursor, putative, expressed	1	Pokkali
Os.14561.1.S1_at	LOC_Os12g42760	Type IIB DNA topoisomerase family protein, expressed	3	Pokkali
Os.14644.1.S1_at	LOC_Os01g74010	Streptomyces cyclase/dehydrase family protein, expressed	8	Pokkali
Os.14783.1.S1_at	LOC_Os04g39170	expressed protein	2	Pokkali
Os.14866.1.S1_at	LOC_Os08g08860	CRS2-associated factor 2, putative, expressed	7	Pokkali
Os.14889.1.S1_at	LOC_Os06g45860	molybdenum cofactor sulfurase, putative, expressed	0	Pokkali
Os.14979.1.S1_at	LOC_Os03g59340	Cellulose synthase A catalytic subunit 3, putative, expressed	9	Pokkali
Os.14985.1.S1_at	LOC_Os02g34630	Myb-like DNA-binding domain containing protein, expressed	10	Pokkali
Os.15.1.S1_at	LOC_Os06g35530	Shaggy-related protein kinase eta, putative, expressed	9	Pokkali
Os.15013.1.S1_at	LOC_Os12g17310	myosin heavy chain, putative, expressed	3	Pokkali
Os.15076.1.S1_at	LOC_Os04g02900	Pyruvate dehydrogenase E1 component alpha subunit, putative, expressed	3	Pokkali
Os.15093.1.S1_at	LOC_Os02g13640	Leucine Rich Repeat family protein, expressed	5	Pokkali
Os.15175.1.S1_at	LOC_Os04g31030	Nitrate-induced NOI protein, expressed	4	Pokkali
Os.15223.1.S1_a_at	LOC_Os08g01130	XPG I-region family protein, expressed	7	Pokkali
Os.15427.1.S1_at	LOC_Os03g64020	expressed protein	6	Pokkali
Os.15571.1.S1_at	LOC_Os12g42230	Pyruvate dehydrogenase E1 component beta subunit, putative, expressed	7	Pokkali
Os.15802.2.S1_x_at	LOC_Os10g31790	ubiquitin family protein, putative, expressed	8	Pokkali
Os.16069.1.S1_at	LOC_Os09g07900	HECT-domain family protein, expressed	6	Pokkali
Os.16079.1.S1_at	LOC_Os05g06260	Gamma-tubulin complex component 4 homolog, putative, expressed	6	Pokkali
Os.16324.1.S1_at	LOC_Os12g38870	expressed protein	7	Pokkali
Os.16530.1.S1_at	LOC_Os01g05420	IWS1 C-terminus family protein, expressed	5	Pokkali
Os.16535.1.S1_at	LOC_Os03g64400	expressed protein	4	Pokkali
Os.16596.1.S1_at	LOC_Os04g37810	expressed protein	7	Pokkali
Os.16627.1.S1_at	LOC_Os03g54750	COBRA-like protein 4 precursor, putative, expressed	10	Pokkali
Os.17120.1.S1_at	LOC_Os02g35070	SPRY domain containing protein, expressed	10	Pokkali
Os.17148.1.A1_at	LOC_Os03g64320	expressed protein	3	Pokkali
Os.17302.1.S1_at	LOC_Os04g31020	expressed protein	3	Pokkali
Os.17436.1.S1_at	LOC_Os04g14510	PHD-finger family protein, expressed	2	Pokkali
Os.17506.1.S1_at	LOC_Os10g32680	Uncharacterized conserved protein, putative, expressed	1	Pokkali
Os.17509.1.S1_at	LOC_Os02g50490	Glycosyl hydrolase family 9 protein, expressed	6	Pokkali
Os.17523.1.S1_at	LOC_Os04g30910	SAM domain family protein, expressed	5	Pokkali
Os.17538.1.S1_at	LOC_Os11g19130	expressed protein	8	Pokkali
Os.17550.1.S1_at	LOC_Os10g25320	Initiation factor 2 subunit family protein, expressed	6	Pokkali
Os.17764.1.S1_at	LOC_Os05g42100	expressed protein	9	Pokkali
Os.17861.1.S2_at	LOC_Os03g51020	protein kinase, putative, expressed	3	Pokkali
Os.17880.1.S1_a_at	LOC_Os08g39860	Non-cyanogenic beta-glucosidase precursor, putative, expressed	7	Pokkali

Os.17902.1.S1_at	LOC_Os05g02420	expressed protein	0	Pokkali
Os.17938.1.A1_s_at	LOC_Os02g51840	expressed protein	10	Pokkali
Os.17969.1.S1_at	LOC_Os02g42200	transcription factor, putative, expressed	10	Pokkali
Os.17981.1.S1_at	LOC_Os02g39920	AT hook motif family protein, expressed	2	Pokkali
Os.17994.1.S1_at	LOC_Os04g41300	ribosome recycling factor family protein, expressed	7	Pokkali
Os.18028.1.S1_at	LOC_Os03g41612	ribosomal 5S rRNA E-loop binding protein Ctc/L25/TL5 containing protein, expressed	6	Pokkali
Os.18074.1.A1_at	LOC_Os02g17250	expressed protein	7	Pokkali
Os.18327.1.S1_at	LOC_Os03g04000	AMP-binding enzyme family protein, expressed	10	Pokkali
Os.18495.1.S1_at	LOC_Os02g16880	membrane protein, putative, expressed	9	Pokkali
Os.18527.1.S1_at	LOC_Os04g08310	expressed protein	7	Pokkali
Os.18663.1.S1_at	LOC_Os09g37230	protein kinase family protein, putative, expressed	0	Pokkali
Os.18745.1.S1_at	LOC_Os03g60880	Permease I, putative, expressed	5	Pokkali
Os.18891.1.S1_at	LOC_Os03g60350	expressed protein	7	Pokkali
Os.18899.1.S1_at	LOC_Os03g14980	expressed protein	7	Pokkali
Os.189.1.S1_at	LOC_Os08g43160	TCP family transcription factor containing protein, expressed	5	Pokkali
Os.18930.1.S1_at	LOC_Os02g43080	salt-inducible protein, putative	3	Pokkali
Os.18975.1.S1_at	LOC_Os02g08370	Ubiquitin carboxyl-terminal hydrolase, family 1 protein, expressed	6	Pokkali
Os.19059.1.S1_at	LOC_Os09g33670	Zinc finger, C3HC4 type family protein, expressed	6	Pokkali
Os.19115.1.S1_at	LOC_Os01g37760	Glutamate/Leucine/Phenylalanine/Valine dehydrogenase family protein, expressed	10	Pokkali
Os.19126.1.S1_at	LOC_Os03g63940	protein kinase AKINbetagamma-2, putative, expressed	7	Pokkali
Os.19281.1.S1_at	LOC_Os02g34690	Transmembrane 9 superfamily protein member 3 precursor, putative, expressed	0	Pokkali
Os.19299.1.S1_at	LOC_Os03g59680	PAPA-1-like conserved region family protein, expressed	2	Pokkali
Os.19367.1.S1_at	LOC_Os03g50980	Sumoylation ligase E3, putative, expressed	4	Pokkali
Os.19412.1.S1_at	LOC_Os01g61640	NLI interacting factor-like phosphatase family protein, expressed	3	Pokkali
Os.19626.1.S1_at	LOC_Os02g39600	outer envelope protein, putative, expressed	9	Pokkali
Os.19872.1.S1_at	LOC_Os02g38240	rhodanese family protein, putative, expressed	10	Pokkali
Os.20083.1.S1_at	LOC_Os08g01390	Phosphatidylinositol-4-phosphate 5-Kinase family protein, expressed	9	Pokkali
Os.20246.1.S1_at	LOC_Os10g32444	expressed protein	3	Pokkali
Os.20258.1.S1_at	LOC_Os12g42150	transducin family protein, putative, expressed	7	Pokkali
Os.20293.1.S1_at	LOC_Os03g59020	T-complex protein 1, theta subunit, putative, expressed	10	Pokkali
Os.20415.1.S1_at	LOC_Os01g01800	expressed protein	10	Pokkali
Os.20521.1.S2_at	LOC_Os03g54040	50S ribosomal protein L6, putative, expressed	4	Pokkali
Os.20562.1.S1_a_at	LOC_Os10g35560	expressed protein	2	Pokkali
Os.20570.1.S1_x_at	LOC_Os06g02500	Superoxide dismutase, chloroplast, putative, expressed	1	Pokkali
Os.20793.1.S1_at	LOC_Os02g34080	myosin family protein, putative, expressed	7	Pokkali
Os.20989.1.S1_at	LOC_Os04g34620	expressed protein	7	Pokkali
Os.21255.1.S1_x_at	LOC_Os08g44050	INDETERMINATE-related protein 1, putative, expressed	10	Pokkali
Os.21320.1.S1_at	LOC_Os04g37470	NADH dependent 6'-deoxychalcone synthase, putative, expressed	10	Pokkali
Os.21415.1.S1_at	LOC_Os12g06100	TLD family protein, expressed	2	Pokkali
Os.21430.2.S1_x_at	LOC_Os08g01420	PHD-finger family protein, expressed	3	Pokkali
Os.22245.1.S1_at	LOC_Os04g32350	RNA polymerase III RPC4 family protein, expressed	10	Pokkali

Os.22379.1.S1_at	LOC_Os04g39240	expressed protein	10	Pokkali
Os.2243.1.S1_a_at	LOC_Os05g03610	phospholipase C, putative, expressed	9	Pokkali
Os.2247.1.S1_at	LOC_Os10g32820	60S ribosomal protein L21, putative, expressed	4	Pokkali
Os.22590.1.A1_at	LOC_Os03g17850	Glycosyltransferase family 43 protein, expressed	9	Pokkali
Os.22592.1.S1_a_at	LOC_Os04g37780	Core histone H2A/H2B/H3/H4 family protein, expressed	7	Pokkali
Os.22598.1.S1_at	LOC_Os04g34430	transposon protein, putative, unclassified, expressed	6	Pokkali
Os.22619.1.S1_at	LOC_Os04g35190	F-box domain containing protein, expressed	9	Pokkali
Os.22699.1.S1_at	LOC_Os03g64140	expressed protein	6	Pokkali
Os.22981.1.S1_at	LOC_Os03g53980	expressed protein	0	Pokkali
Os.23196.1.S1_x_at	LOC_Os03g61950	Kelch motif family protein, expressed	9	Pokkali
Os.23410.1.A1_at	LOC_Os07g09690	galactosyltransferase family protein, putative, expressed	6	Pokkali
Os.24174.1.A1_at	LOC_Os08g38240	transposon protein, putative, CACTA, En/Spm sub-class, expressed	3	Pokkali
Os.24183.1.A1_at	LOC_Os06g13860	Poly polymerase catalytic domain containing protein, expressed	8	Pokkali
Os.24311.1.A1_at	LOC_Os06g12876	expressed protein	5	Pokkali
Os.2444.1.S1_at	LOC_Os11g10100	mitogen-activated kinase kinase kinase alpha, putative, expressed	6	Pokkali
Os.24620.1.S1_at	LOC_Os08g06344	transposon protein, putative, CACTA, En/Spm sub-class, expressed	3	Pokkali
Os.24639.1.S1_at	LOC_Os03g25620	Low temperature viability protein, expressed	9	Pokkali
Os.24827.1.S1_at	LOC_Os04g41910	RNA recognition motif family protein, expressed	3	Pokkali
Os.24912.1.S1_a_at	LOC_Os02g11820	GTPase activating protein, putative, expressed	8	Pokkali
Os.25091.1.A1_at	LOC_Os12g15420	Pre-mRNA splicing factor cwc22, putative, expressed	5	Pokkali
Os.25298.1.S1_at	LOC_Os07g48370	Glycosyl transferase family 8 protein, expressed	4	Pokkali
Os.25462.1.S1_at	LOC_Os03g63290	oxidoreductase, short chain dehydrogenase/reductase family protein, expressed	3	Pokkali
Os.25589.3.S1_x_at	LOC_Os11g25800	PB1 domain containing protein, expressed	1	Pokkali
Os.25983.1.S1_a_at	LOC_Os03g17010	RNA and export factor-binding protein, putative, expressed	5	Pokkali
Os.26040.1.S1_at	LOC_Os03g62630	ribosomal protein S6 family protein, putative, expressed	7	Pokkali
Os.26047.2.S1_s_at	LOC_Os08g39420	expressed protein	2	Pokkali
Os.26079.1.S1_at	LOC_Os02g50600	glycosyl transferase family 8 protein, putative, expressed	2	Pokkali
Os.2614.1.S1_at	LOC_Os03g04260	Glutathione S-transferase, N-terminal domain containing protein, expressed	0	Pokkali
Os.26475.1.S1_at	LOC_Os03g57500	zinc finger family protein, putative, expressed	3	Pokkali
Os.26512.1.S1_at	LOC_Os03g58350	AUX/IAA family protein, expressed	1	Pokkali
Os.26648.1.S1_at	LOC_Os10g02760	hydroxyproline-rich glycoprotein family protein, putative, expressed	7	Pokkali
Os.2673.1.S1_at	LOC_Os03g50290	14-3-3-like protein S94, putative, expressed	0	Pokkali
Os.26778.1.S1_at	LOC_Os04g22360	expressed protein	1	Pokkali
Os.26779.1.S1_at	LOC_Os05g07960	F-box protein interaction domain containing protein, expressed	4	Pokkali
Os.26784.1.S1_a_at	LOC_Os12g42730	Mov34/MPN/PAD-1 family protein, expressed	7	Pokkali
Os.26831.1.A1_a_at	LOC_Os02g43906	ceramide kinase, putative, expressed	9	Pokkali
Os.26860.1.S1_at	LOC_Os08g38410	RNA recognition motif family protein, expressed	1	Pokkali
Os.26902.1.S1_at	LOC_Os03g45970	expressed protein	3	Pokkali
Os.26955.1.S1_at	LOC_Os08g39150	expressed protein	7	Pokkali
Os.27073.1.S1_at	LOC_Os01g57550	nodulation protein, putative, expressed	5	Pokkali
Os.27124.1.S1_at	LOC_Os02g10290	Copper-transporting ATPase 3, putative, expressed	10	Pokkali

Os.27125.1.A1_at	LOC_Os04g54820	Programmed cell death protein 2, C-terminal domain containing protein, expressed	7	Pokkali
Os.27757.1.A1_s_at	LOC_Os10g23220	expressed protein	6	Pokkali
Os.27772.1.S1_x_at	LOC_Os06g20320	trigger factor family protein, expressed	5	Pokkali
Os.27772.2.S1_at	LOC_Os12g34860	phenylalanyl-tRNA synthetase class IIc family protein, putative, expressed	5	Pokkali
Os.27784.1.S1_at	LOC_Os09g37012	Eukaryotic aspartyl protease family protein, expressed	2	Pokkali
Os.27874.2.S1_x_at	LOC_Os05g48840	expressed protein	10	Pokkali
Os.27955.1.S2_at	LOC_Os03g63270	regulatory protein, putative, expressed	10	Pokkali
Os.27981.1.S1_at	LOC_Os03g59700	Peptidyl-prolyl cis-trans isomerase H, putative, expressed	10	Pokkali
Os.28088.1.S1_at	LOC_Os03g58050	40S ribosomal protein S18, putative, expressed	6	Pokkali
Os.28213.1.S1_at	LOC_Os12g41600	expressed protein	4	Pokkali
Os.28255.1.S1_at	LOC_Os04g54790	ABC1 family protein, expressed	6	Pokkali
Os.28397.1.S1_at	LOC_Os02g41650	Phenylalanine ammonia-lyase, putative, expressed	4	Pokkali
Os.28405.1.S1_x_at	LOC_Os03g55389	Casein kinase II, alpha chain 1, putative, expressed	9	Pokkali
Os.28405.2.S1_at	LOC_Os03g58240	guanylate-binding family protein, putative, expressed	10	Pokkali
Os.2952.1.S1_x_at	LOC_Os03g25270	pyrrolidone-carboxylate peptidase family protein, expressed	8	Pokkali
Os.31147.1.S1_at	LOC_Os03g06610	allergen, putative, expressed	10	Pokkali
Os.31151.2.S1_at	LOC_Os03g59180	expressed protein	10	Pokkali
Os.3196.1.S1_at	LOC_Os10g32920	60S ribosomal protein L23, putative, expressed	2	Pokkali
Os.32454.1.S1_at	LOC_Os01g01650	Isoflavone reductase homolog IRL, putative, expressed	4	Pokkali
Os.32482.1.S1_at	LOC_Os01g59090	thylakoid lumenal 20 kDa protein, putative, expressed	10	Pokkali
Os.32782.1.S1_at	LOC_Os08g43170	Hydroxymethylglutaryl-CoA synthase, putative, expressed	6	Pokkali
Os.33176.1.S1_at	LOC_Os07g44970	transcription-coupled DNA repair protein, putative, expressed	4	Pokkali
Os.33271.2.S1_x_at	LOC_Os08g06200	Stomatin-like protein 2, putative, expressed	0	Pokkali
Os.3383.1.S1_a_at	LOC_Os03g54084	Phytochrome C, putative, expressed	10	Pokkali
Os.33874.2.A1_x_at	LOC_Os07g44830	proline-rich family protein, putative, expressed	9	Pokkali
Os.33939.1.A1_at	LOC_Os01g04590	expressed protein	4	Pokkali
Os.33966.1.S1_at	LOC_Os11g32270	expressed protein	5	Pokkali
Os.33983.1.S1_at	LOC_Os01g09790	IQ calmodulin-binding motif family protein, expressed	3	Pokkali
Os.34006.1.S1_x_at	LOC_Os08g16350	expressed protein	9	Pokkali
Os.3409.1.S1_at	LOC_Os03g59040	Squalene synthetase, putative, expressed	6	Pokkali
Os.34146.1.S1_at	LOC_Os03g61080	expressed protein	10	Pokkali
Os.34979.1.S1_at	LOC_Os05g32060	expressed protein	3	Pokkali
Os.35230.1.S1_at	LOC_Os01g04130	peptidyl-tRNA hydrolase family protein, expressed	4	Pokkali
Os.35753.1.S1_a_at	LOC_Os07g11060	expressed protein	2	Pokkali
Os.37365.1.S1_at	LOC_Os02g43519	expressed protein	6	Pokkali
Os.37576.1.S1_at	LOC_Os03g60130	transcription elongation factor S-II family protein, expressed	10	Pokkali
Os.37882.1.S1_at	LOC_Os09g32976	60S ribosomal protein L7a, putative, expressed	8	Pokkali
Os.38014.1.S1_at	LOC_Os01g14100	folate/biopterin transporter family protein, expressed	1	Pokkali
Os.38192.1.S1_at	LOC_Os07g14160	Polygalacturonase family protein, expressed	1	Pokkali
Os.38273.2.S1_x_at	LOC_Os04g24414	expressed protein	6	Pokkali
Os.38330.1.A1_at	LOC_Os01g56000	RNA recognition motif family protein, expressed	7	Pokkali

Os.38734.1.S1_s_at	LOC_Os09g13610	phytochrome and flowering time regulatory protein, putative, expressed	0	Pokkali
Os.39128.1.A1_at	LOC_Os06g34810	hypothetical protein	0	Pokkali
Os.3933.1.S1_a_at	LOC_Os03g28400	Trafficking protein particle complex subunit 3, putative, expressed	8	Pokkali
Os.40415.1.A1_s_at	LOC_Os05g49060	expressed protein	4	Pokkali
Os.4124.1.S1_a_at	LOC_Os01g51390	Insulinase containing protein, expressed	3	Pokkali
Os.4149.2.S1_x_at	LOC_Os03g50810	Protein kinase domain containing protein, expressed	9	Pokkali
Os.415.1.S1_at	LOC_Os10g32970	Eukaryotic translation initiation factor 4E-2, putative, expressed	6	Pokkali
Os.4196.1.S1_at	LOC_Os01g10020	Type I phosphodiesterase/nucleotide pyrophosphatase family protein, expressed	7	Pokkali
Os.4421.1.S1_at	LOC_Os06g01490	Monocopper oxidase precursor, putative, expressed	8	Pokkali
Os.4482.1.A1_at	LOC_Os01g13090	expressed protein	0	Pokkali
Os.45412.1.S1_at	LOC_Os01g04690	hypothetical protein	7	Pokkali
Os.4609.1.S1_a_at	LOC_Os10g35870	Membrane steroid binding protein 1, putative, expressed	8	Pokkali
Os.46271.1.S1_at	LOC_Os12g38670	transposon protein, putative, CACTA, En/Spm sub-class	5	Pokkali
Os.46507.1.S1_at	LOC_Os10g39980	transmembrane protein, putative, expressed	2	Pokkali
Os.46557.1.S1_x_at	LOC_Os05g41940	transposon protein, putative, CACTA, En/Spm sub-class, expressed	1	Pokkali
Os.46562.1.S1_at	LOC_Os10g32870	ribosomal protein L11 containing protein, expressed	3	Pokkali
Os.46776.1.S1_s_at	LOC_Os09g26960	Cytochrome P450 family protein, expressed	4	Pokkali
Os.4698.1.S1_at	LOC_Os01g70770	Glutathione S-transferase III, putative, expressed	8	Pokkali
Os.47921.1.A1_at	LOC_Os08g39050	pentatricopeptide, putative, expressed	10	Pokkali
Os.47980.1.S1_at	LOC_Os04g08764	Avr9/Cf-9 rapidly elicited protein 137, putative, expressed	10	Pokkali
Os.4807.1.S1_at	LOC_Os04g54200	diacylglycerol kinase, putative, expressed	10	Pokkali
Os.48184.1.A1_at	LOC_Os07g39750	Esterase precursor, putative, expressed	10	Pokkali
Os.48315.1.S1_at	LOC_Os03g61800	glycoside hydrolase family 28 protein, putative, expressed	4	Pokkali
Os.48351.1.S1_at	LOC_Os03g50140	Plastocyanin-like domain containing protein, expressed	9	Pokkali
Os.48368.1.S1_s_at	LOC_Os06g04560	armadillo/beta-catenin repeat family protein, putative, expressed	8	Pokkali
Os.48429.1.S1_at	LOC_Os03g63970	Gibberellin 20 oxidase 1, putative, expressed	7	Pokkali
Os.48518.1.S1_at	LOC_Os01g67740	SMC proteins Flexible Hinge Domain containing protein, expressed	5	Pokkali
Os.4857.1.S1_at	LOC_Os03g62090	Cellulose synthase A catalytic subunit 6, putative, expressed	8	Pokkali
Os.48600.1.A1_s_at	LOC_Os09g10820	Oxidoreductase family, NAD-binding Rossmann fold containing protein, expressed	3	Pokkali
Os.48611.1.S1_at	LOC_Os03g64010	expressed protein	5	Pokkali
Os.48856.1.S1_at	LOC_Os08g04110	Phosphate/phosphoenolpyruvate translocator, putative, expressed	10	Pokkali
Os.49004.1.S1_at	LOC_Os12g30060	KE2 family protein, expressed	6	Pokkali
Os.49020.1.A1_at	LOC_Os03g03880	Serine/threonine-protein kinase RLCKVII, putative, expressed	1	Pokkali
Os.49083.1.S1_x_at	LOC_Os09g36670	Peptidyl-prolyl cis-trans isomerase 3, putative, expressed	9	Pokkali
Os.49110.1.S1_at	LOC_Os06g14060	BTB/POZ domain containing protein, expressed	7	Pokkali
Os.49230.1.S1_at	LOC_Os03g04450	expressed protein	9	Pokkali
Os.49281.1.S1_s_at	LOC_Os06g21240	Glycine rich protein family protein, expressed	6	Pokkali
Os.49397.1.S1_at	LOC_Os04g14450	pentatricopeptide, putative, expressed	6	Pokkali
Os.4943.1.S1_at	LOC_Os02g43870	Protein kinase domain containing protein, expressed	7	Pokkali
Os.4961.1.S1_a_at	LOC_Os02g52230	TRANSPORT INHIBITOR RESPONSE 1 protein, putative, expressed	6	Pokkali
Os.4961.3.S1_x_at	LOC_Os02g52230	TRANSPORT INHIBITOR RESPONSE 1 protein, putative, expressed	3	Pokkali

Os.49675.1.S1_at	LOC_Os05g26820	CBL-interacting serine/threonine-protein kinase 15, putative, expressed	9	Pokkali
Os.49897.1.S1_at	LOC_Os08g10430	NB-ARC domain containing protein, expressed	2	Pokkali
Os.4990.1.S1_at	LOC_Os05g05470	T-complex protein 1, zeta subunit, putative, expressed	0	Pokkali
Os.49945.1.S1_at	LOC_Os09g36810	Glycosyl hydrolases family 35 protein, expressed	10	Pokkali
Os.50155.1.S1_at	LOC_Os06g12960	expressed protein	3	Pokkali
Os.5027.1.S2_at	LOC_Os01g52864	integral membrane family protein, putative, expressed	7	Pokkali
Os.50398.1.S1_at	LOC_Os02g44090	expressed protein	9	Pokkali
Os.50460.1.S1_at	LOC_Os03g59620	patatin, putative, expressed	1	Pokkali
Os.50488.1.S1_at	LOC_Os02g13950	NHL repeat-containing protein, putative, expressed	2	Pokkali
Os.50534.1.S1_at	LOC_Os04g33530	Ser/Thr protein phosphatase family protein, expressed	9	Pokkali
Os.50631.1.S1_at	LOC_Os10g31970	SNF2 domain-containing protein, putative, expressed	0	Pokkali
Os.50871.1.S1_at	LOC_Os02g50560	DEAD/DEAH box helicase, putative, expressed	10	Pokkali
Os.51093.1.S1_at	LOC_Os02g42580	BRO1-like domain containing protein, expressed	8	Pokkali
Os.51394.1.S1_at	LOC_Os05g07890	Embryo-specific protein 3, containing protein, expressed	9	Pokkali
Os.51409.1.S1_at	LOC_Os03g55630	expressed protein	4	Pokkali
Os.51616.1.S1_at	LOC_Os03g16600	expressed protein	3	Pokkali
Os.51702.1.S1_at	LOC_Os01g59570	Protein kinase domain containing protein, expressed	3	Pokkali
Os.51717.1.S1_at	LOC_Os02g39390	expressed protein	7	Pokkali
Os.51729.1.S1_at	LOC_Os02g02570	autophagy protein Apg5 family, putative, expressed	8	Pokkali
Os.51832.1.S1_at	LOC_Os02g42412	expressed protein	5	Pokkali
Os.5206.1.S1_at	LOC_Os02g51790	50S ribosomal protein L29, chloroplast precursor, putative, expressed	5	Pokkali
Os.52111.1.S1_at	LOC_Os05g11870	coenzyme A diphosphatase NUDT11, putative, expressed	3	Pokkali
Os.52203.1.S1_at	LOC_Os12g36100	kinesin motor protein, putative, expressed	7	Pokkali
Os.52212.1.S1_at	LOC_Os08g05670	HEAT repeat family protein, expressed	5	Pokkali
Os.52233.1.S1_at	LOC_Os01g02890	Phosphatidyl serine synthase family protein	5	Pokkali
Os.52287.2.S1_at	LOC_Os02g02810	expressed protein	0	Pokkali
Os.52470.1.S1_at	LOC_Os03g48190	expressed protein	9	Pokkali
Os.52509.1.S1_at	LOC_Os04g33950	Transcription factor E2F/dimerisation partner family protein, expressed	7	Pokkali
Os.52573.1.S1_at	LOC_Os09g36580	Thaumatin family protein, expressed	10	Pokkali
Os.52610.1.S1_at	LOC_Os01g73060	expressed protein	2	Pokkali
Os.52642.1.S1_at	LOC_Os06g01460	expressed protein	3	Pokkali
Os.52754.1.S1_x_at	LOC_Os03g50230	Protein kinase domain containing protein, expressed	7	Pokkali
Os.52758.1.S1_at	LOC_Os02g02580	expressed protein	10	Pokkali
Os.52928.1.S1_at	LOC_Os02g51810	chloroplast outer membrane translocon subunit, putative, expressed	5	Pokkali
Os.53083.1.S1_at	LOC_Os09g01930	hypothetical protein	7	Pokkali
Os.5311.1.S1_at	LOC_Os08g39630	Helix-loop-helix DNA-binding domain containing protein, expressed	4	Pokkali
Os.5312.1.S1_at	LOC_Os09g12570	Ubiquitin-conjugating enzyme spm2, putative, expressed	8	Pokkali
Os.53212.1.S1_at	LOC_Os04g21660	26S protease regulatory subunit 6A homolog, putative, expressed	3	Pokkali
Os.53301.1.S1_at	LOC_Os02g16660	ATPase, AAA family protein, expressed	9	Pokkali
Os.5351.1.S1_at	LOC_Os03g61890	AIG1 family protein, expressed	7	Pokkali
Os.53598.1.S1_at	LOC_Os07g48360	ATP-dependent DNA helicase, RecQ family protein, expressed	1	Pokkali

Os.53603.1.S1_at	LOC_Os12g42700	expressed protein	2	Pokkali
Os.53964.1.S1_at	LOC_Os12g36170	G14587-6, putative, expressed	7	Pokkali
Os.53982.1.S1_at	LOC_Os05g41890	expressed protein	8	Pokkali
Os.5399.1.S1_at	LOC_Os06g50310	bZIP transcription factor family protein, expressed	8	Pokkali
Os.54039.1.S1_at	LOC_Os02g03590	expressed protein	6	Pokkali
Os.54078.1.S1_at	LOC_Os02g42300	expressed protein	8	Pokkali
Os.54110.1.S1_at	LOC_Os02g09530	expressed protein	9	Pokkali
Os.54125.1.S1_at	LOC_Os09g38620	NADPH-cytochrome P450 reductase, putative, expressed	6	Pokkali
Os.54230.1.S1_at	LOC_Os04g11790	F-box domain containing protein, expressed	10	Pokkali
Os.54293.1.S1_at	LOC_Os03g11510	Mitochondrial carrier protein, expressed	7	Pokkali
Os.54305.1.S1_at	LOC_Os06g12610	Auxin efflux carrier component 1, putative, expressed	2	Pokkali
Os.54356.1.S1_at	LOC_Os01g67960	GPI transamidase component PIG-S, putative, expressed	4	Pokkali
Os.54360.1.S1_at	LOC_Os03g44530	expressed protein	8	Pokkali
Os.54394.1.S1_at	LOC_Os02g52460	Zinc knuckle family protein, expressed	0	Pokkali
Os.54426.1.S1_at	LOC_Os04g22860	Protein phosphatase inhibitor containing protein, expressed	2	Pokkali
Os.54484.2.S1_at	LOC_Os06g04080	Glycosyl hydrolases family 17 protein, expressed	2	Pokkali
Os.54498.1.S1_at	LOC_Os04g32940	Leucine Rich Repeat family protein, expressed	4	Pokkali
Os.54593.1.S1_at	LOC_Os08g43430	pepper esterase, putative, expressed	5	Pokkali
Os.54688.1.S1_at	LOC_Os12g39090	expressed protein	6	Pokkali
Os.5493.1.S1_at	LOC_Os08g03870	expressed protein	6	Pokkali
Os.55011.1.S1_x_at	LOC_Os01g20120	expressed protein	0	Pokkali
Os.5507.1.S1_at	LOC_Os12g07010	60S ribosomal protein L3, putative, expressed	8	Pokkali
Os.55287.1.S1_at	LOC_Os05g15180	expressed protein	9	Pokkali
Os.55303.1.S1_at	LOC_Os02g01700	hypothetical protein	10	Pokkali
Os.55640.1.S1_at	LOC_Os02g37000	Mitochondrial prohibitin complex protein 1, putative, expressed	8	Pokkali
Os.55696.1.S1_at	LOC_Os12g39100	expressed protein	10	Pokkali
Os.56279.1.S1_at	LOC_Os08g02540	Adenylate kinase family protein, expressed	6	Pokkali
Os.56352.1.S1_at	LOC_Os06g01470	LOC569692 protein, putative, expressed	6	Pokkali
Os.5642.1.S1_at	LOC_Os03g58620	small multi-drug export protein, putative, expressed	2	Pokkali
Os.56528.1.S1_at	LOC_Os05g42210	S-locus glycoprotein family protein, expressed	10	Pokkali
Os.5675.1.S1_at	LOC_Os03g60110	KH domain-containing protein, putative, expressed	10	Pokkali
Os.57411.1.S1_at	LOC_Os04g33650	expressed protein	7	Pokkali
Os.57430.1.S1_at	LOC_Os02g08530	Protein kinase domain containing protein, expressed	4	Pokkali
Os.57534.1.S1_x_at	LOC_Os08g43090	bZIP protein, putative, expressed	4	Pokkali
Os.5803.1.S1_at	LOC_Os04g33510	expressed protein	1	Pokkali
Os.5869.1.S1_a_at	LOC_Os04g38410	Chlorophyll a-b binding protein CP24 10B, chloroplast precursor, putative, expressed	1	Pokkali
Os.5901.1.S1_at	LOC_Os12g29550	expressed protein	7	Pokkali
Os.6001.1.S1_at	LOC_Os10g30910	transmembrane protein, putative, expressed	4	Pokkali
Os.6042.1.S1_at	LOC_Os07g25810	transposon protein, putative, CACTA, En/Spm sub-class, expressed	6	Pokkali
Os.6065.1.S1_at	LOC_Os04g35900	translocon Tic40, putative, expressed	3	Pokkali
Os.6093.1.S1_a_at	LOC_Os02g10700	Leucine Rich Repeat family protein, expressed	8	Pokkali

Os.6122.1.S1_at	LOC_Os08g40110	N-acyl-L-amino-acid amidohydrolase family protein, expressed	7	Pokkali
Os.6176.1.S1_at	LOC_Os08g43370	Glucosamine-6-phosphate isomerase/6-phosphogluconolactonase family protein,	8	Pokkali
Os.6265.1.S1_at	LOC_Os12g34850	Fibronectin type III domain containing protein, expressed	10	Pokkali
Os.6349.1.S1_a_at	LOC_Os03g60780	Armadillo/beta-catenin-like repeat family protein, expressed	8	Pokkali
Os.6361.1.S1_at	LOC_Os02g16620	reticulon family protein, putative, expressed	10	Pokkali
Os.6428.1.S1_at	LOC_Os01g10340	expressed protein	10	Pokkali
Os.6501.1.A1_at	LOC_Os08g43180	expressed protein	8	Pokkali
Os.6548.1.S1_at	LOC_Os02g42600	C-terminal domain phosphatase-like 1, putative, expressed	0	Pokkali
Os.6634.1.S1_at	LOC_Os02g41590	Adenosine kinase 2, putative, expressed	2	Pokkali
Os.6741.1.S1_at	LOC_Os02g39300	LUC7 N_terminus domain-containing protein, putative, expressed	6	Pokkali
Os.6764.2.S1_at	LOC_Os04g54300	wound induced protein, putative, expressed	2	Pokkali
Os.6807.1.S1_at	LOC_Os11g24560	Sec23/Sec24 trunk domain containing protein, expressed	2	Pokkali
Os.6828.1.S1_at	LOC_Os03g59220	Ubiquinol-cytochrome c reductase complex 14 kDa protein, putative, expressed	9	Pokkali
Os.6854.1.S1_at	LOC_Os09g36040	Rhodanese-like domain containing protein, expressed	8	Pokkali
Os.6881.1.S1_at	LOC_Os03g61700	expressed protein	7	Pokkali
Os.6933.1.S1_at	LOC_Os04g41920	expressed protein	8	Pokkali
Os.7114.1.S1_s_at	LOC_Os03g15780	anthranilate synthase component I family protein, expressed	9	Pokkali
Os.7153.1.S1_at	LOC_Os12g34880	expressed protein	10	Pokkali
Os.7166.1.S1_at	LOC_Os03g60610	CRAL/TRIO domain containing protein, expressed	10	Pokkali
Os.7169.1.S1_at	LOC_Os09g39034	expressed protein	7	Pokkali
Os.7234.1.S1_at	LOC_Os03g58840	Vesicle-associated membrane protein 724, putative, expressed	1	Pokkali
Os.7283.2.S1_x_at	LOC_Os03g58590	RE14858p, putative, expressed	0	Pokkali
Os.7318.2.S1_at	LOC_Os09g27370	Spc97/Spc98 family protein, expressed	2	Pokkali
Os.7326.1.S1_at	LOC_Os04g37660	protein phosphatase 2C, putative, expressed	7	Pokkali
Os.7339.1.S1_at	LOC_Os10g31850	CHY zinc finger family protein, expressed	7	Pokkali
Os.7359.1.S1_at	LOC_Os07g45090	NADH-ubiquinone oxidoreductase 51 kDa subunit, mitochondrial precursor, putative, expressed	4	Pokkali
Os.7402.1.S1_at	LOC_Os05g08010	F-box domain containing protein, expressed	5	Pokkali
Os.7406.1.S1_at	LOC_Os08g01840	survival motor neuron domain containing 1, putative, expressed	7	Pokkali
Os.7411.1.S1_at	LOC_Os09g24250	ubiquitin specific protease 39, putative, expressed	10	Pokkali
Os.742.1.S1_x_at	LOC_Os01g04720	leucine-rich repeat protein, putative, expressed	2	Pokkali
Os.7453.1.S1_x_at	LOC_Os10g08940	phosphatidylinositolglycan class O, putative, expressed	10	Pokkali
Os.7453.2.S1_x_at	LOC_Os10g08940	phosphatidylinositolglycan class O, putative, expressed	5	Pokkali
Os.7586.1.S1_at	LOC_Os05g27950	cp protein, putative, expressed	3	Pokkali
Os.7589.1.S1_at	LOC_Os06g46930	50S ribosomal protein L24, chloroplast precursor, putative, expressed	1	Pokkali
Os.7602.1.S1_at	LOC_Os02g44108	Beta-expansin 4 precursor, putative, expressed	5	Pokkali
Os.7612.1.S1_at	LOC_Os03g60840	Bowman-Birk serine protease inhibitor family protein, expressed	7	Pokkali
Os.7624.1.S1_at	LOC_Os03g02390	Mitochondrial import inner membrane translocase subunit Tim17 family protein, expressed	3	Pokkali
Os.7657.1.S1_at	LOC_Os03g60090	Methylenetetrahydrofolate reductase, putative, expressed	3	Pokkali
Os.7657.1.S1_at	LOC_Os03g60090	Methylenetetrahydrofolate reductase, putative, expressed	6	Pokkali
Os.7715.1.S1_a_at	LOC_Os05g41900	Protein translation factor SUI1 homolog, putative, expressed	10	Pokkali
Os.7864.1.S1_at	LOC_Os03g60100	50S ribosomal protein L17, putative, expressed	2	Pokkali

Os.7909.1.S1_at	LOC_Os02g50240	Glutamine synthetase root isozyme 3, putative, expressed	8	Pokkali
Os.7912.1.S1_at	LOC_Os03g55540	Zinc-finger protein 1, putative, expressed	1	Pokkali
Os.8030.1.S1_at	LOC_Os09g19700	Protein kinase APK1A, chloroplast precursor, putative, expressed	6	Pokkali
Os.8104.1.S1_a_at	LOC_Os03g17570	Two-component response regulator-like PRR73, putative, expressed	7	Pokkali
Os.8121.1.S1_at	LOC_Os03g61760	SBP domain containing protein, expressed	10	Pokkali
Os.8142.1.S1_a_at	LOC_Os09g36220	Two-component response regulator-like PRR95, putative, expressed	6	Pokkali
Os.8275.1.S1_at	LOC_Os05g49270	Sugar transporter family protein, expressed	8	Pokkali
Os.8281.1.S1_at	LOC_Os12g41620	transducin family protein, putative, expressed	8	Pokkali
Os.8294.1.S1_at	LOC_Os02g09359	Protein kinase domain containing protein, expressed	3	Pokkali
Os.8343.1.S1_at	LOC_Os06g02570	Syntaxin 43, putative, expressed	7	Pokkali
Os.8401.2.S1_at	LOC_Os06g48350	Eukaryotic translation initiation factor 5, putative, expressed	3	Pokkali
Os.8455.1.S1_at	LOC_Os07g28610	expressed protein	9	Pokkali
Os.8479.1.S1_s_at	LOC_Os04g34460	plastid-lipid associated protein PAP, putative, expressed	8	Pokkali
Os.8521.1.S1_at	LOC_Os07g11110	mRNA-binding protein precursor, putative, expressed	9	Pokkali
Os.8542.1.S1_at	LOC_Os12g37590	prefoldin subunit 3, putative, expressed	9	Pokkali
Os.8549.1.S1_at	LOC_Os03g50130	MAPEG family protein, expressed	5	Pokkali
Os.8570.2.S1_x_at	LOC_Os08g39820	expressed protein	1	Pokkali
Os.8586.1.S1_at	LOC_Os03g57780	leucine-rich repeat transmembrane protein kinase, putative, expressed	2	Pokkali
Os.8600.1.S1_at	LOC_Os04g37480	NADH-dependent oxidoreductase 1, putative, expressed	10	Pokkali
Os.8686.1.S1_at	LOC_Os03g57840	Sterol-regulatory element binding protein site 2 protease containing protein, expressed	3	Pokkali
Os.8707.1.S1_at	LOC_Os03g62730	transposon protein, putative, unclassified, expressed	2	Pokkali
Os.8707.2.A1_at	LOC_Os03g62740	GDSL-motif lipase/hydrolase family protein, putative, expressed	4	Pokkali
Os.8707.3.S1_x_at	LOC_Os03g62740	GDSL-motif lipase/hydrolase family protein, putative, expressed	10	Pokkali
Os.8737.1.S1_at	LOC_Os08g29730	expressed protein	6	Pokkali
Os.8767.1.S1_x_at	LOC_Os07g08330	60S ribosomal protein L4, putative, expressed	10	Pokkali
Os.8792.1.S1_at	LOC_Os12g38930	Glycosyl transferase family 8 protein, expressed	3	Pokkali
Os.9010.4.S1_x_at	LOC_Os03g63910	pentatricopeptide, putative, expressed	8	Pokkali
Os.9015.1.S1_at	LOC_Os01g73980	Caricain precursor, putative, expressed	5	Pokkali
Os.9024.1.S1_at	LOC_Os02g38190	expressed protein	3	Pokkali
Os.9075.1.S1_at	LOC_Os03g60400	40S ribosomal protein S23, putative, expressed	8	Pokkali
Os.9077.1.S1_at	LOC_Os10g41940	expressed protein	10	Pokkali
Os.9183.1.S1_at	LOC_Os03g59060	Serine/threonine protein phosphatase PP2A catalytic subunit, putative, expressed	1	Pokkali
Os.9251.1.S1_at	LOC_Os10g35960	NAD-dependent malic enzyme 59 kDa isoform, mitochondrial precursor, putative, expressed	10	Pokkali
Os.9355.1.S1_at	LOC_Os01g72490	lateral root primordium 1, putative, expressed	10	Pokkali
Os.9367.1.S1_a_at	LOC_Os09g16910	Cysteine desulfurase, mitochondrial precursor, putative, expressed	10	Pokkali
Os.9502.1.S1_at	LOC_Os03g54910	expressed protein	3	Pokkali
Os.9520.1.S1_at	LOC_Os08g08830	expressed protein	10	Pokkali
Os.9526.1.S1_at	LOC_Os12g06260	Harpin-induced protein 1 containing protein, expressed	4	Pokkali
Os.9630.1.S1_at	LOC_Os04g35290	Thioredoxin family protein, expressed	9	Pokkali
Os.9639.1.S1_at	LOC_Os02g35039	NAD dependent epimerase/dehydratase family protein, expressed	8	Pokkali
Os.9729.1.S1_a_at	LOC_Os02g17390	Peroxisomal fatty acid beta-oxidation multifunctional protein, putative, expressed	10	Pokkali

Os.9742.1.S1_at	LOC_Os09g12560	Ferrochelatase II, chloroplast precursor, putative, expressed	6	Pokkali
Os.9781.2.S1_x_at	LOC_Os01g39800	Esterase, putative, expressed	8	Pokkali
Os.9922.1.S1_s_at	LOC_Os08g16010	acetyltransferase, GNAT family protein, expressed	10	Pokkali
OsAffx.10921.1.S1_x_at	LOC_Os01g05500	zinc finger in N-recognin family protein, expressed	9	Pokkali
OsAffx.11878.1.S1_s_at	LOC_Os02g03530	expressed protein	6	Pokkali
OsAffx.11895.1.S1_at	LOC_Os02g04970	expressed protein	0	Pokkali
OsAffx.12446.1.S1_at	LOC_Os02g42040	Up-frameshift suppressor 2 family protein, expressed	8	Pokkali
OsAffx.13491.1.S1_at	LOC_Os03g55180	expressed protein	6	Pokkali
OsAffx.15212.1.S1_at	LOC_Os06g01320	'chromo' domain containing protein, expressed	3	Pokkali
OsAffx.15770.1.S1_at	LOC_Os06g36070	expressed protein	5	Pokkali
OsAffx.15784.1.S1_at	LOC_Os06g36800	MBOAT family protein	8	Pokkali
OsAffx.15786.1.S1_at	LOC_Os06g36910	Rf1 protein, mitochondrial precursor, putative	4	Pokkali
OsAffx.18018.1.S1_s_at	LOC_Os09g36920	La domain containing protein, expressed	5	Pokkali
OsAffx.19516.1.S1_at	LOC_Os12g06150	uncharacterized plant-specific domain TIGR01568 family protein, expressed	10	Pokkali
OsAffx.19600.1.S1_x_at	LOC_Os07g45920	transposon protein, putative, CACTA, En/Spm sub-class	9	Pokkali
OsAffx.22672.1.S1_at	LOC_Os03g47650	TPR Domain containing protein, expressed	6	Pokkali
OsAffx.23124.1.S1_at	LOC_Os01g05430	Rhomboid family protein, expressed	2	Pokkali
OsAffx.23222.1.S1_s_at	LOC_Os01g13080	60S acidic ribosomal protein P3, putative, expressed	6	Pokkali
OsAffx.24187.1.S1_at	LOC_Os02g10490	expressed protein	7	Pokkali
OsAffx.24292.1.S1_at	LOC_Os02g17460	Tesmin/TSO1-like CXC domain containing protein	5	Pokkali
OsAffx.24704.1.S1_s_at	LOC_Os02g43560	WRKY family transcription factor, putative, expressed	1	Pokkali
OsAffx.2480.1.S1_at	LOC_Os02g01760	diphosphomevalonate decarboxylase family protein	4	Pokkali
OsAffx.25115.1.S1_at	LOC_Os03g17050	expressed protein	5	Pokkali
OsAffx.25707.1.S1_at	LOC_Os03g53920	Kinesin motor domain containing protein, expressed	8	Pokkali
OsAffx.26329.1.S1_x_at	LOC_Os04g34620	expressed protein	8	Pokkali
OsAffx.29587.1.S1_s_at	LOC_Os08g38300	Histone H2B, putative, expressed	8	Pokkali
OsAffx.30158.1.S1_at	LOC_Os09g36760	expressed protein	3	Pokkali
OsAffx.31686.1.S1_s_at	LOC_Os12g10650	expressed protein	1	Pokkali
OsAffx.31710.1.S1_x_at	LOC_Os12g12260	Diacylglycerol kinase 1, putative, expressed	4	Pokkali
OsAffx.3200.1.S1_s_at	LOC_Os03g11400	Targeting protein for Xklp2 containing protein, expressed	10	Pokkali
OsAffx.4002.1.S1_s_at	LOC_Os04g35580	expressed protein	5	Pokkali
OsAffx.4139.1.S1_s_at	LOC_Os04g50720	RWD domain containing protein, expressed	4	Pokkali
OsAffx.4236.1.S1_at	LOC_Os05g02400	RNA recognition motif family protein, expressed	1	Pokkali
OsAffx.4296.1.S1_at	LOC_Os05g10930	Glutaredoxin-like family protein, expressed	6	Pokkali
OsAffx.5683.1.S1_x_at	LOC_Os08g02540	Adenylate kinase family protein, expressed	5	Pokkali
OsAffx.6086.1.S1_at	LOC_Os08g39740	expressed protein	2	Pokkali
Os.10370.2.S1_x_at	unknown		1	IR29
Os.12943.1.S1_at	unknown		2	IR29
Os.14832.1.S1_at	unknown		7	IR29
Os.14923.2.S1_at	unknown		3	IR29
Os.18305.1.S2_at	unknown		10	IR29

Os.25521.2.S1_x_at	unknown	10	IR29
Os.27210.1.S1_at	unknown	8	IR29
Os.50660.1.S1_at	unknown	8	IR29
Os.51465.1.S1_x_at	unknown	6	IR29
Os.53062.1.S2_at	unknown	10	IR29
Os.5412.1.S1_at	unknown	5	IR29
Os.55282.1.S1_x_at	unknown	10	IR29
Os.5720.1.S2_at	unknown	3	IR29
Os.6014.2.S1_x_at	unknown	6	IR29
Os.9167.1.A1_at	unknown	9	IR29
Os.9899.1.S2_at	unknown	5	IR29
OsAffx.11866.1.S1_at	unknown	10	IR29
OsAffx.15921.1.S1_at	unknown	4	IR29
OsAffx.4094.1.S1_x_at	unknown	0	IR29
Os.12794.1.A1_at	unknown	1	Pokkali
Os.24620.1.S2_a_at	unknown	10	Pokkali
Os.25983.1.S2_at	unknown	4	Pokkali
Os.3439.1.S1_a_at	unknown	7	Pokkali
Os.47943.1.A1_at	unknown	2	Pokkali
Os.49109.1.S2_at	unknown	3	Pokkali
Os.7316.1.S1_at	unknown	6	Pokkali
OsAffx.30318.1.S1_at	unknown	8	Pokkali
