# Supplementary material for: Detection and validation of single feature polymorphisms using RNA expression data from a rice genome array
Source: BMC Plant Biol. 2009 May 29;9:65. doi: 10.1186/1471-2229-9-65 (PMC2697985; doi:10.1186/1471-2229-9-65)
Supplement: Additional file 2 — Sequenced SFP probe sets and the information of each SFP position. The data show the information including gene models, chromosome numbers of sequenced SFP probe sets, and nucleotide sequences at SNP or INDEL of each SFP position. [file 1471-2229-9-65-S2.pdf]

Additional file 2
Sequenced SFP probe sets and the information of each SFP position

Probe set name	Gene model	Chromosome No	Physical position (5' end)	Pokkali [a]	IR29 [a]	FL478 [a]	Saltol [b]
OsAffx.27908.1.S1_at	LOC_Os01g12860.1	1	7113923	T	G	G	
Os.4482.1.A1_at	LOC_Os01g13090.3	1	7278814	ACT	---	ACT	
Os.38014.1.S1_at	LOC_Os01g14100.3	1	7897352	C	T	C	
Os.874.1.S1_at	LOC_Os01g14610.2	1	8179897	A	G	G	
Os.38074.1.S1_x_at	LOC_Os01g14780.1	1	8266390	A,A,G,A,-	G,C,-,C,T	G,C,-,C,T	
Os.14625.1.S1_at	LOC_Os01g14810.2	1	8272276	T	A	A	
Os.10666.1.S1_at	LOC_Os01g15260.1	1	8537445	T	A	A	
Os.10662.1.S1_at	LOC_Os01g15320.1	1	8574725	GT	CC	CC	
Os.9029.1.S1_at	LOC_Os01g15350.1	1	8596216	AAAC	----	----	
Os.34400.1.S1_at	LOC_Os01g15770.1	1	8880280	A	C	C	
Os.94.1.S1_at	LOC_Os01g15979.1	1	8985713	T	C	C	
Os.455.1.S1_at	LOC_Os03g20370.1	1	9192919	G	A	A	Saltol
Os.37639.1.S1_at	LOC_Os01g16414.4	1	9320117	C	G	G	Saltol
Os.37842.1.S1_at	LOC_Os01g16520.1	1	9374978	A	G	G	Saltol
Os.247.1.S1_at	LOC_Os01g16650.1	1	9442463	-----	CACCT	CACCT	Saltol
Os.14702.1.S1_a_at	LOC_Os01g17020.1	1	9746901	G	A	A	Saltol
Os.7948.1.S1_a_at	LOC_Os01g17150.1	1	9856128	C	A	A	Saltol
Os.55011.1.S1_x_at	LOC_Os01g20120.1	1	11427774	A	G	A	Saltol
Os.45751.1.A1_x_at	LOC_Os01g20880.1	1	11637965	G	C	C	Saltol
Os.13500.2.S1_x_at	LOC_Os01g20940.2	1	11676292	T	C	C	Saltol
Os.35123.1.S1_at	LOC_Os01g22230.1	1	12482404	A	G	G	Saltol
Os.24895.1.S1_at	LOC_Os01g24060.1	1	13543313	G	T	T	Saltol
Os.33510.1.S2_at	LOC_Os01g25320.1	1	14285672	A	C	C	Saltol
Os.25255.1.S1_at	LOC_Os01g25530.1	1	14454283	G	A	A	Saltol
Os.18293.1.S1_at	LOC_Os01g26020.1	1	14734782	A	G	G	Saltol
Os.40545.1.S1_x_at	LOC_Os01g26160.2	1	14792157	G	A	A	Saltol
Os.12845.1.S1_at	LOC_Os01g26920.1	1	15324971	T	C	C	Saltol
Os.4023.1.S1_at	LOC_Os01g27020.1	1	15386316	T	G	G	Saltol
OsAffx.23435.1.S1_at	LOC_Os01g28730.1	1	16413616	G	A	A	

Os.27455.1.S1_x_at	LOC_Os01g29820.1	1	17034358	C	T	T
Os.28207.1.S1_at	LOC_Os01g34200.1	1	19180422	T	C	C
Os.11689.1.S1_at	LOC_Os01g34330.1	1	19250456	G	T	T
Os.7133.1.S1_at	LOC_Os01g34700.1	1	19462649	C	T	T
Os.47996.1.A1_at	LOC_Os01g36390.1	1	20518897	T	A	A
Os.10115.1.S1_at	LOC_Os01g36460.1	1	20569587	--	GT	GT
Os.33852.1.S1_at	LOC_Os01g36630.1	1	20649545	G	A	A
Os.19115.1.S1_at	LOC_Os01g37760.1	1	21445766	C	T	C
Os.38249.1.S1_at	LOC_Os01g38530.2	1	21969952	G	A	A
Os.33723.1.S1_at	LOC_Os01g49950.1	1	29026743	T	C	C
Os.13960.2.S1_x_at	LOC_Os01g73560.1	1	42948552	G	A	G
Os.14644.1.S1_at	LOC_Os01g74010.1	1	43199534	T	C	T
Os.6741.1.S1_at	LOC_Os02g39300.1	2	23723421	C	T	C
Os.22967.1.S1_s_at	LOC_Os03g02070.2	3	636130	GA	--	--
Os.18327.1.S1_at	LOC_Os03g04000.1	3	1800611	G	T	G
Os.7114.1.S1_s_at	LOC_Os03g15780.1	3	8686829	T	C	T
Os.52202.1.S1_at	LOC_Os03g22450.1	3	12848021	T	C	C
Os.8686.1.S1_at	LOC_Os03g57840.1	3	32890478	A	T	A
Os.8707.2.A1_at	LOC_Os03g62730.1	3	35445756	G	A	G
Os.48429.1.S1_at	LOC_Os03g63970.1	3	36089654	CATC,G	----,A	CATC,G
Os.18527.1.S1_at	LOC_Os04g08310.1	4	4424010	-,C	G,A	-,C
Os.8814.1.S1_at	LOC_Os04g11880.1	4	6491586	T	C	C
Os.14326.1.S1_at	LOC_Os04g35920.2	4	21717379	T	A	T
Os.49160.1.S1_at	LOC_Os04g40300.1	4	23744231	-	T	T
Os.7865.1.S1_at	LOC_Os06g15400.1	6	8751607	T	C	C
Os.9234.1.S1_at	LOC_Os07g46540.1	7	27797803	T	A	A
Os.7085.2.S1_x_at	LOC_Os09g24200.1	7	28493024	A	C	C
Os.52921.1.S1_at	LOC_Os08g14760.1	8	8867836	G	A	A
Os.14078.1.S1_s_at	LOC_Os10g07210.1	10	3771545	AT	--	AT
Os.17533.1.S1_at	LOC_Os11g08950.1	11	4734761	C	T	T
Os.18880.1.S1_at	LOC_Os11g23170.3	11	12853173	C	G	G
Os.5713.1.S1_at	LOC_Os11g31650.1	11	18007057	T	C	C
Os.5412.1.S1_at	unknown	unknown	unknown	G	T	T
Os.116.1.S1_at	LOC_Os01g15610.1	1	8767676	ALL THE SAME SEQ		

Os.3655.1.S1_at	LOC_Os01g18744.1	1	10562090	-----	-----	-----	Saltol

[a] nucleotide at SNP or INDEL in the SFP position of each probe set; -, INDEL

[b] Saltol, probe sets in Saltol region
