# Supplementary material for: Detection and validation of single feature polymorphisms using RNA expression data from a rice genome array
Source: BMC Plant Biol. 2009 May 29;9:65. doi: 10.1186/1471-2229-9-65 (PMC2697985; doi:10.1186/1471-2229-9-65)
Supplement: Additional file 3 — Primer list and amplicon lengths of sequenced SFP-containing probe sets. The data represent primer sequences for amplicon sequencing of the SFP-containing probe sets and their amplicon lengths. [file 1471-2229-9-65-S3.pdf]

Additional file 3

Primer list and amplicon lengths of sequenced SFP-containing probe sets

Affymetrix probe set (or Gene model)	Forward primer (5'->3')	Reverse primer (5'->3')	Amplicon length	
			Expected size by Primer3 (bp)	Real size (kb) in agarose gel
Probe sets in Saltol region				
Os.247.1.S1_at	ACATCCTCCAGGACAAGTGG	GGAGCACAAGGGAGCAATTA	520	0.55
Os.455.1.S1_at	TGAGTAATGGCTGCGTTCTG	TCCCGTTGGAAACTCTGATT	780	0.75
Os.3655.1.S1_at	GATGATACGGGAGGCGAAG	CTGGGTCACCAAGTGAACTTAT	552	0.55
Os.4023.1.S1_at	AAGCAGCTCCAGAAGGACAA	CAATAGCGTGGCAAAAGGTT	642	0.65
Os.7948.1.S1_a_at	ACTGGCAAAGGGTGACATTC	TCTTCTGGACAAGCCTCCTG	572	0.55
Os.12845.1.S1_at	AGCAACACGTTGTGAACCAG	TGACATAGGCTGTGGACCAA	542	0.55
Os.13500.2.S1_x_at	AGAGCATCAATCAACCTCTG	TTCATCTCAGGTCGCTTGTG	499	0.5
Os.14702.1.S1_a_at	TTTTCCCCACTTGCAGTACC	ACTCCAAATTCAACGGATGC	504	0.5
Os.18293.1.S1_at	CTGGTTTCGGATGAGGAATG	CGAGGAGATACTGGTAACTG	344	0.35
Os.24895.1.S1_at	ATACTGGGTGCAGGAGGATG	CCCACTGGGTTCTCATGATT	705	0.7
Os.25255.1.S1_at	GAGACGCTTGATGACGATGA	CTACCAAAGTTGCCGAAAGC	624	0.6
Os.33510.1.S2_at	ATTTGCTTCCTGGACTGGTG	CTCTCCCAAAATGGAATGTTT	483	0.5
Os.35123.1.S1_at	CAGATCAGGGTCAACTGCAA	TGTACGGGAGGAAGGAGATG	552	0.55
Os.37639.1.S1_at	GGAACTTCGTCCTCTTGTGC	GTGTGGCGTACACAGGTCAG	712	0.73
Os.37842.1.S1_at	ATGCGGAATCTCAAACAAGG	TCGTTGGATGTGATGATGCT	455	0.45
Os.40545.1.S1_x_at	GATTTGTGGCAATGCAGGAT	GCTGCTGAAGTAAGCCTGGT	569	0.55
Os.45751.1.A1_x_at	TTTCCCACCCCTATCATTCA	TTAGCAAAGACGCCAGATGA	661	0.65
Os.55011.1.S1_x_at	GATCCATTGCCTCACAGGTT	AAAACGCAGGGAACTTTCAC	561	0.55
LOC_Os01g19220	ACACCAACTTCACCTACGCGTC	GCACATCACAGGCCATCTGTGTC	889	0.9
LOC_Os01g19400	TTCTCCCTATGATGCAGAGGAAG	GGTAGGATTTGTTGTTTCACACAAGTG	387	0.4
LOC_Os01g20160 (*SKC1*)	TATAAATGGCATGTTCAATTCG	TGTGTTAAAAATAAACTGTGTCAA	852	0.85
Other probe sets				
Os.94.1.S1_at	GTCCTTTGCCCACACAACTT	ACCATGCCACACCCTATCAT	595	0.6
Os.116.1.S1_at	TGGTCATTAAATTGCCTCTTC	GTCAGATTTGGTACATCTCG	477	0.57

Os.874.1.S1_at	GGCTGCTCAGATTGTGTTGA	GCACCGTTGTTACGAAACTATTC	601	0.6
Os.4482.1.A1_at	CGCTAAAATTGCCATCCTCT	AAACCGCAGTTTCATCCTTG	403	0.43
Os.9029.1.S1_at	TGAAATGGAACAGGGGTCAT	ACATCCAAACTCGGACCAAA	449	0.45
Os.10662.1.S1_at	GGGTACATCGGGTACGACG	CCAAGCAGCATAGCAAACAA	361	0.35
Os.10666.1.S1_at	CGATTCTAAAGCCGTCTTGC	CACCCACCAAATTCAATTCC	631	0.6
Os.14625.1.S1_at	TGCGGTCGTTTTCTTCTTTC	ACAAATTGTTTTCGGCTGCT	303	0.35
Os.34400.1.S1_at	GCTGGTGAGTGCCTACTGGT	GGTTTACCTGGTTGGGGTTT	495	0.5
Os.38014.1.S1_at	AGTAAGATCACACCTATTACC	AGGGCAAAGAAAACAAAAGCA	877	0.9
Affx.23435.1.S1_at	AGGGATATCAAAGCGAGCAA	GGTGGGGGCAAAAGTACAAT	757	0.75
Os.7133.1.S1_at	GCTTGCCATGTGCCTAACTT	ACGCCAGATGACTTGAGCTT	688	0.7
Os.10115.1.S1_at	CGACTTCCTCTGACCTCTCG	ATGTGTGCCCAACAAAACAA	630	0.6
Os.11689.1.S1_at	CGCATCGTCATTAGGGAGTT	AAAGGGGCACAACAGCATAC	586	0.6
Os.19115.1.S1_at	GGTTTCAACCCAGAAGCTGA	GCCTACCATTTTGTGCGTTT	812	0.8
Os.28207.1.S1_at	TTCTTTGCGGGGAAGTATTG	TAAAAAGGGACCGAGGGAGT	666	0.65
Os.33723.1.S1_at	GGACACGAACCCAATAATGC	CATCCTCAGCTCGATCATCA	689	0.7
Os.33852.1.S1_at	TGTTGGGTTTCTCATTTCAGG	GATGAAGGTTGCGATACCAGA	689	0.7
Os.38249.1.S1_at	GCAAAGCATTTTTGGAAAGC	GGAAACAGAACATGGGCATT	685	0.7
Os.47996.1.A1_at	CGGGACAATCTCGTTGCAG	TGTGTAGAACAAATACAAATATG	619	0.65
Os.5412.1.S1_at	CATCCCATGTCCCATGTGTA	CATCCCATTTTGCCATTCAAG	304	0.3
Os.5713.1.S1_at	GCCTTTTCTGACATCCTTGC	TAGCATGACAAGCCAAACGA	573	0.6
Os.6741.1.S1_at	AACTGAAGGCACCAGGTTCA	TGGGTTTCAAGAGGAGAGGA	603	0.6
Os.7085.2.S1_x_at	AGAAGAAGCAGGAGCAGCAG	CGACAACAAATCAAGGTCCA	513	0.55
Os.7114.1.S1_s_at	GCTTATCGCTCTTGCACTCC	TACATGCCTCCACCAAACAA	452	0.45
Os.7865.1.S1_at	AGATCGCTGGAACGGAGTAA	TCTGAAGGGAGGAGTCATGG	419	0.4
Os.8686.1.S1_at	TTTTTGCCCTCACCTTGAAC	TTGTCGCCATCAACTAGCAG	678	0.7
Os.8707.2.A1_at	GCGGAACAAAGGCAATTAAG	GGAAGCAAATGGTTGTTGGT	509	0.5
Os.8814.1.S1_at	ACAGGCTGATTGCGTAAACC	ATGAACTTCCGATGGTCTGG	628	0.65
Os.9234.1.S1_at	ATCAGACGGTGACAGCACAG	TACACCTAAAACATGTACTCC	853	0.9
Os.13960.2.S1_x_at	TCTGGAGTAACTCTGGATGT	CCCTTCAACTCAAGCTCTGG	528	0.55
Os.14078.1.S1_s_at	CGTGCTCGTCATCAAGAAGA	CATGCAGGCACAAGTGAAAG	514	0.55
Os.14326.1.S1_at	CCCAAGGTTGCTATTCTGGA	GAGATTAAGGCACCCAACGA	571	0.55
Os.14644.1.S1_at	ATGGGCGAAGAAAGATGATG	TGTGTCAAAAGTGCGAAACC	752	0.75
Os.17533.1.S1_at	GAGGACCATGCCAACAAAGT	TGTGCCACTTCTCATCCAAG	543	0.55

Os.18327.1.S1_at	TACCAATCCCGCAATTTCTC	ACAAGGTCGTCCTGATACGC	578	0.6
Os.18527.1.S1_at	TGAGACGGTTGGCAATATGA	ACGCATGTTCATCTGTTTCG	563	0.6
Os.18880.1.S1_at	GTCGGCATCCAATTAGGAAA	GGCTCGTACCCTGATAGCTG	446	0.45
Os.22967.1.S1_s_at	TGGTTCGTCCTTGTGTTTCA	AAGTTGCCCACCACATTCTC	688	0.7
Os.27455.1.S1_x_at	AGTTTTTGGATCGTCATCACC	CAGCTCAACTCGAAAGAAAG	562	0.55
Os.38074.1.S1_x_at	GAGTCTCAGGTGGGCATCAT	GGCAGCCAGTGAAGTGGATA	329	0.35
Os.48429.1.S1_at	GGAGATGGACACGGTGGTG	TATAGATCATAGATGGGTACG	459	0.45
Os.49160.1.S1_at	CATGGCTCTAGTTCGTTTTTC	CCTGCACGCTTTGATACAGA	675	0.7
Os.52202.1.S1_at	CAACACCGCACTACATGGAC	ACAAGCCATACAACGCATGA	572	0.6
Os.52921.1.S1_at	AAATACCAGTCGCCTTCGTG	TTCTCGCCCCTTCTCTAACA	669	0.65
OsAffx.27908.1.S1_at	TTTTCCAAGCCTGGATGTTC	AGTCATTCAGGGCATTAACTT	670	0.7
